# Von der Geschichtsphilosophie zur Formalpragmatik: Zur Geschichte und Gegenwart Kritischer Theorie

**DOI:** 10.1007/s42048-021-00106-7

**Published:** 2021-07-20

**Authors:** Sven Ellmers

**Affiliations:** Institut für Philosophie, Fakultät IV, Carl von Ossietzky Universität Oldenburg, 26111 Oldenburg, Deutschland

**Keywords:** Kritische Theorie, Frankfurter Schule, Dialektik der Aufklärung, Horkheimer, Adorno, Habermas, Critical Theory, Frankfurt School, Dialectic of Enlightenment, Horkheimer, Adorno, Habermas

## Abstract

Die gegenwärtige Debatte über die normativen Grundlagen Kritischer Theorie lässt sich nur vor dem Hintergrund ihrer Geschichte verstehen: Die Defizite in den Konzeptionen der 1. und 2. Generation strukturieren das Feld der Optionen, die heute noch als aussichtsreich gelten können. Der Blick zurück schärft den Blick nach vorn. Im ersten Teil des Beitrags werde ich deshalb die Hauptthesen, ethischen Implikationen und Einseitigkeiten von Horkheimers *Zur Kritik der instrumentellen Vernunft* darstellen. Im zweiten Teil werde ich die (von Horkheimer beiläufig angedachte und) von Habermas ausgearbeitete These diskutieren, dass der Sprache das nicht-instrumentelle Telos der Verständigung innewohnt. Zudem werde ich zeigen, dass es Habermas nicht gelingt, den für seine Diskursethik zentralen Universalisierungsgrundsatz abzuleiten. Wie sich die Begründungsprobleme bei Horkheimer und Habermas auf die gegenwärtige Debatte auswirken, werde ich im dritten Teil besprechen.

## Abstract (en)

The current debate about the normative foundations of critical theory can only be understood in the light of its history: The deficits in the concepts of the 1st and 2nd generation structure the field of options that can still be considered promising today. Looking back sharpens the view ahead. In the first part of the paper, therefore, I will present the main theses, ethical implications and one-sidedness of Horkheimer’s *Eclipse of Reason*. In the second part, I will discuss the assumption of Habermas, that the non-instrumental telos of understanding is inherent in language. Moreover, I will show that Habermas does not succeed in deriving the principle of universalization that is central to his discourse ethics. In the third part I will discuss, how the justification problems at Horkheimer and Habermas affect the current debate.

Er nennt’s Vernunft und braucht’s allein,

Nur tierischer als jedes Tier zu sein.

Goethe, *Faust*

## Horkheimers Kritik instrumenteller Vernunft

### Die Hauptthesen

Das argumentative Grundgerüst von *Zur Kritik der instrumentellen Vernunft* findet sich im dritten Kapitel, das mit ‚Die Revolte der Natur‘ überschrieben ist. Aus diesem Kapitel stammt die folgende Passage, die ich Schritt für Schritt interpretieren werde:

[1] Der Mensch teilt im Prozeß seiner Emanzipation das Schicksal seiner übrigen Welt. Naturbeherrschung schließt Menschenbeherrschung ein. [2] Jedes Subjekt hat nicht nur an der Unterjochung der äußeren Natur, der menschlichen und der nicht-menschlichen, teilzunehmen, sondern muß, um das zu leisten, die Natur in sich selbst unterjochen. [3] Herrschaft wird um der Herrschaft willen ‚verinnerlicht‘. [4] Was gewöhnlich als Ziel bezeichnet wird – das Glück des Individuums, Gesundheit und Reichtum –, gewinnt seine Bedeutung ausschließlich von seiner Möglichkeit, funktional zu werden. (ZK 106)

Ad 1: Der Mensch, so Horkheimer, kehrt das ursprüngliche Verhältnis zur Natur um. War er ihr früher weitgehend ausgeliefert, spanne er sie dank technischer Fortschritte zunehmend für sich ein. Diese Emanzipation verändere zugleich die sozialen Beziehungen: Naturbeherrschung schließe Menschenbeherrschung ein. Wie ist das zu verstehen? Die Aussage liest sich wie eine *Subjunktion*: Sobald Menschen beginnen, sich die Natur zu unterwerfen, etablieren sie unweigerlich auch Strukturen sozialer Herrschaft. Schon rein sprachlich liegt diese Deutung nahe: Wenn man sagt, A schließe B ein, meint man für gewöhnlich eine materiale Implikation. Im vorliegenden Fall wäre sie allerdings begründungsbedürftig. Warum soll es ausgeschlossen sein, die äußere Natur in egalitär-kooperativer Weise zu beherrschen? Falls Horkheimer diese Möglichkeit tatsächlich für ausgeschlossen hält, bedürfte es eines stützenden Arguments – eines Arguments, das eine Brücke von der Naturbeherrschung zur Menschenbeherrschung schlägt. Dafür wiederum müsste Horkheimer zunächst seinen Begriff der Naturbeherrschung präzisieren. Naturbeherrschung kann entweder in einem ganz basalen und unproblematischen Sinn bedeuten, dass der Mensch zum Zwecke seiner Selbsterhaltung und Bedürfnisbefriedigung darauf angewiesen ist, kraft seiner eigenen Natur auf die ihm äußerliche Natur einzuwirken. Von diesem basalen Begriff ausgehend lässt sich jedoch kein notwendiger Zusammenhang zur sozialen Herrschaft herstellen – und täte man es doch, wäre die Überwindung sozialer Herrschaft nicht länger denkbar. Für Horkheimer kann folglich nicht bereits der technische Umgang mit der Natur Grund allen Übels sein: nicht „die Technik oder das Motiv der Selbsterhaltung an sich“ seien das Problem, „nicht die Produktion *per se*“ (ZK 157). Mit Naturbeherrschung ist folglich eine über die *conditio humana* hinausgehende, radikal instrumentelle wie zerstörerische Naturbeziehung gemeint. Das Brückenargument könnte dann lauten, dass diese Form der Naturbeziehung die Charakterdispositionen der Menschen auf eine so grundlegende Art und Weise prägt, dass die zwischenmenschlichen Beziehungen davon nicht unberührt bleiben können. Tatsächlich argumentiert Horkheimer an späterer Stelle genau in diesem Sinne: „Diese Form der Wahrnehmung [gemeint ist die Wahrnehmung der Natur als auszubeutendes Objekt, S.E.] hat auch die Weise bestimmt, in der die Menschen in ihren ökonomischen und politischen Verhältnissen sich ein Bild voneinander machen. Die Muster, nach denen die Menschheit die Natur anschaut, wirken schließlich zurück auf die Spiegelung von Menschen im menschlichen Geist, determinieren sie“ (ZK 119).

Ad 2: Jedes Subjekt muss sich selbst unterjochen, um die äußere Natur und andere Menschen unterjochen zu können. Vor dem Hintergrund der Erläuterungen zu [1] ließe sich präzisieren: Horkheimer meint nicht *jedes* (nur denkbare) Subjekt, sondern nur die bisherigen Subjekte heteronom verfasster Gesellschaften; mit „unterjochen“ meint er nicht das unerlässliche Maß an Selbstkontrolle und Bedürfnisaufschub, sondern eine darüber hinausgehende Selbstbeschneidung, ja Selbstverstümmelung des Subjekts.^1^

Ad 3: Dieser Satz fügt auf den ersten Blick nichts Neues hinzu, sondern stellt eine Wiederholung von [2] dar: Um die Natur und Menschen als (bloße) Objekte behandeln zu können, bedarf es auch einer objektivierenden Einstellung gegenüber sich selbst.

Ad 4: Horkheimer nutzt die Mehrdeutigkeit von [3], um die *Selbstzweckhaftigkeit von Herrschaft* zu behaupten. Der Satz „Herrschaft wird um der Herrschaft willen ‚verinnerlicht‘“ wird nun nicht mehr im Sinne von [2] ausgelegt (‚Herrschaft wird verinnerlicht, um Natur und Menschen beherrschen zu können‘), sondern die Selbstunterjochung wird als derart radikal unterstellt, dass selbst die Zwecke, die ursprünglich die Herrschaft über Natur und Andere begründeten („Glück des Individuums, Gesundheit und Reichtum“) zu bloßen Mitteln eines universellen/verselbständigten Herrschafts- oder Machtstrebens degradiert werden. Die „Unterjochung der Natur innerhalb und außerhalb des Menschen“ gehe „ohne ein sinnvolles Motiv vonstatten“ (ebd., 106). Entsprechend heißt es auch in der *Dialektik der Aufklärung*: „Das Mittel wird fetischisiert: es absorbiert die Lust.“ „Herrschaft überlebt als Selbstzweck […]. Genuß zeigt schon die Spur des Veralteten“ (DdA 124). Die „Beherrschung der Natur drinnen und draußen“ wurde „zum absoluten Lebenszweck gemacht“ (DdA 49).

Hier deutet sich bereits an, was Horkheimer mit seiner Genealogie instrumenteller Weltbeziehungen vor Augen zu schweben scheint: die von Marx entlehnte Figur des Kapitalverhältnisses als „automatisches Subjekt“, das ebenfalls nicht mehr auf menschliche Zwecke bezogen ist, sondern in einem sinnfreien wie endlosen Prozess nur noch seine eigene Verwertung forciert. Gleichwohl ist Horkheimers Projekt nur bedingt an Marx orientiert. Als Marx in seiner Kritik der politischen Ökonomie darlegte, dass das Kapitalverhältnis sich gegenüber allen Klassen verselbständigt hat, sodass noch die materiell profitierenden Kapitalisten lediglich Anhängsel eines selbstzweckhaften, durch die Konkurrenz der Privateigentümer angetriebenen Akkumulationsprozesses sind, betonte er stets die Differenz zu vorbürgerlichen Gesellschaften. Ihm wäre es nicht in den Sinn gekommen, Odysseus – eine Heldenfigur aus dem 8.-7. Jahrhundert vor Christus – als „Urbild […] des bürgerlichen Individuums“ (DdA 61) zu porträtieren. Vorbürgerliche Gesellschaften beruhten nach Marx auf gänzlich anderen Prinzipien. In ihnen reproduzierten sich soziale Ungleichheiten nicht *systemisch* unter Bedingungen der *Rechtsgleichheit*. Sie beruhten im Gegenteil auf rechtlichen Privilegien, die auch von den Beherrschten als begründet akzeptiert wurden – den Ägyptern erschienen die Privilegien der Pharaonen als legitim, weil sie die Pharaonen für göttlich hielten (vgl. Godelier 2000, 161 ff.) –, sich zur Not durch physische Gewalt aufrechterhalten ließen und letztlich der Aneignung von Konsumtionsgütern dienten. Zudem war die auf bestimmte Wirtschaftszweige begrenzte Warenproduktion noch durch nicht-ökonomische Institutionen und Überzeugungen geprägt – sie folgte keiner Eigenlogik, war nicht „disembedded“ (Karl Polanyi). Für Horkheimers kulturanthropologische Überlegungen sind diese Differenzen gerade nicht entscheidend. Wohlgemerkt besteht der Gegensatz zwischen Horkheimer und Marx nicht darin, dass jener nur Kontinuitäten und dieser nur Diskontinuitäten in der menschlichen Geschichte gesehen hätte. Das Band, welches Marx zufolge alle bisherigen Gesellschaften verbindet, ist das der Ausbeutung: Aneignung fremder Arbeit. Anders als Horkheimer (und Adorno^2^) verankert Marx diese Herrschaft von Menschen über Menschen jedoch nicht in einer vorgängigen Unterjochung der Natur durch den Menschen. In seiner Kapitalismusanalyse kommt Marx vielmehr zum gegenteiligen Schluss: Es ist die Spezifik der zwischenmenschlichen Verhältnisse der Moderne, die eine die Naturressourcen zerstörende Akkumulationsspirale in Gang setzt.^3^

Der unmittelbare Anlass, eine mit dem Naturverhältnis ansetzende Universalgeschichte zu schreiben, war eine Erschütterung des marxistischen Hintergrundkonsenses, der das Institut in den 1930er-Jahren noch auszeichnete. Zwar war bereits die Programmatik eines interdisziplinären Materialismus von dem Gedanken getragen, die marxsche Ökonomiekritik müsse neu interpretiert, aktualisiert und um andere wissenschaftliche Disziplinen erweitert werden (vor allem durch die Psychoanalyse Freuds), gegen Ende der 1930er-Jahren kommt Horkheimer jedoch zu dem Schluss, dass der bisherige theoretische Rahmen nicht mehr ausreicht, die Katastrophen der Gegenwart zu begreifen. Um mit ihnen mitzuhalten, bedarf es aus seiner Sicht eines philosophischen Neuanfangs. Das Resultat ist eine Abkehr vom Marxismus – insbesondere von der Annahme, die stetige Produktivkraftentwicklung münde letztlich in Autonomie –, die starke Überschneidungen mit Nietzsches *Genealogie der Moral* aufweist. Dies betrifft nicht nur das genealogische Verfahren, sondern auch die Trias von Naturherrschaft, Selbstdisziplin und sozialer Herrschaft. Der zivilisierte Mensch, so Nietzsche, könne nicht mehr auf seine Triebe zurückgreifen, die ihn früher, als er noch ein freier Wilder war, sicher geleitet hätten. Er muss sie fortan unterdrücken, sich zusammennehmen, ihnen entsagen. Als Folge davon ist er nun auf das „Denken, Schließen, Berechnen, Kombinieren von Ursachen und Wirkungen reduziert“. Allerdings „hatten jene alten Instinkte nicht mit einem Male aufgehört, ihre Forderungen zu stellen.“ Ihre Energien flossen zum einen in den Aufbau eines inneren Seelenreichs, zum anderen wandten sich die zivilisatorisch gehemmten Instinkte nun „*gegen den Menschen selbst*“: Es entsteht „die Feindschaft, die Grausamkeit, die Lust an der Verfolgung, am Überfall, am Wechsel, an der Zerstörung“ (Nietzsche 1995, 825). Des Weiteren ist die Abkehr vom Marxismus in erheblichem Maße noch von diesem selbst inspiriert:

1. Es ist auffällig, dass Horkheimer weiterhin bestrebt ist, materialistisch zu argumentieren, nimmt seine Genealogie doch ihren Ausgangspunkt in der Selbsterhaltung des Menschen.

2. Das Interesse an einer materialen Geschichtsphilosophie war in der marxistischen Tradition weit verbreitet. Man denke nur an den Historischen Materialismus, den, so könnte man sagen, Horkheimer mit einem negativen Vorzeichen versieht. In *Zur Kritik der instrumentellen Vernunft* wird sowohl das „causa-sui-Konzept der Produktivkräfte“ (Ritsert 1988, 69) als auch das Basis-Überbau-Schema beibehalten. So wird der Siegeszug instrumenteller Weltbeziehungen mit der „Entwicklung der Produktionsmethoden“ (ZK 113) erklärt und das Zustandekommen „rechtlicher, künstlerischer und religiöser Formen“ auf die „Waffen- und Maschinenarten“ zurückgeführt, „die der Mensch auf den verschiedenen Stufen seiner Entwicklung benutzt“ (ZK 114). Es stellt sich allerdings die Frage, *warum* die instrumentelle Rationalität im Zuge der Produktivkraftentwicklung zunimmt. Anstelle einer Antwort finden sich lediglich Andeutungen: „Indem die materielle Produktion und soziale Organisation komplizierter und verdinglichter werden, wird es immer schwieriger, die Mittel als solche zu erkennen, da sie die Erscheinung autonomer Wesenheiten annehmen.“ (ZK 113) Offenbar ist Horkheimer der Ansicht, dass die Komplexität und Emergenz von moderner Technik und sozialen Institutionen so weit fortgeschritten ist, dass die Menschen sie gar nicht mehr als Mittel, sondern, wie im frühen Mythos, als selbständige Entitäten auffassen – Entitäten, die nicht das Vermögen praktischer Vernunft (Zwecksetzungskompetenz) fördern, sondern zur Unterwerfung anhalten. Die erste (äußere) Natur wird also nur dadurch gebrochen, dass eine zweite (technisch-institutionelle) Natur geschaffen wird, die am Ende ebenso undurchschaubar und unverfügbar ist, wie es die erste Natur zu Beginn der Menschheitsgeschichte war. Der geschichtsphilosophische Impuls lag aber auch der von Engels begründeten und im Institut für richtig befundenen^4^ logisch-historischen Interpretation des *Kapitals* sowie den Schriften Walter Benjamins zugrunde.

3. Noch die Figur der selbstzweckhaften Kapitalverwertung kehrt in Gestalt einer Herrschaft um der Herrschaft willen wieder: Die Menschheitsgeschichte habe von Beginn an im Zeichen einer expandierenden Vernunft gestanden, die keine Zwecke an sich mehr kenne (wie „Glück des Individuums, Gesundheit und Reichtum“), sondern buchstäblich *alles* zum Mittel herabwürdige. Während Aristoteles die Vorstellung noch für abwegig hielt, dass *jeder* Zweck nur Mittel für einen anderen Zweck sei (und es folglich kein oberstes Gut gebe), geht Horkheimer offenbar davon aus, dass der infinite Regress der Instrumentalisierung Realität geworden ist. Die Bemerkung von Habermas, Horkheimer habe mit instrumenteller Vernunft die „zur Totalität aufgespreizte Zweckrationalität“ (Habermas 1985, 144) diagnostiziert, trifft es daher nur bedingt. Wenn Vernunft sich laut Horkheimer „damit begnügen muss, *alles* auf ein bloßes Werkzeug zu reduzieren“, weshalb „ihr *einziges* verbleibendes Ziel die Perpetuierung ihrer gleichschaltenden Tätigkeit“ (ZK 105, Herv. von mir) sei, handelt es sich nur um eine halbierte Zweckrationalität: Bar jeder Zwecksetzungskompetenz ist die subjektive Vernunft lediglich ein Organ verabsolutierter Mittelrationalität – ein bloßes „Mittel der Mittelsuche“ (Hubig 1981, 165). Die Zwecke dieser Mittelsuche sind entweder vom herrschenden Machtapparat^5^ vorgegeben oder Resultat einer prinzipienlos-dezisionistischen und damit heteronomen Wahl (vgl. ZK 31). Mit Weber, den Horkheimer als prominenten Vertreter der instrumentellen Vernunft nennt, hat dies nur wenig zu tun. Bei Weber ist die Mittelsuche nur ein Teilaspekt der Zweckrationalität, die Ziele, Mittel und Nebenfolgen zueinander in Beziehung setzt. Zweckrational handelt, wer „sowohl die Mittel gegen die Zwecke, wie die Zwecke gegen die Nebenfolgen, wie endlich auch die verschiedenen möglichen Zwecke gegeneinander rational abwägt“ (Weber 1972). Zweckrationalität ist *nicht* identisch mit Instrumentalität (vgl. Hubig 1981).

Abgesehen davon hält Horkheimer seinen radikalen Begriff subjektiver Vernunft nicht konsequent durch, kennt er doch durchaus ein letztes Ziel der Mittelsuche, nämlich das der *Selbsterhaltung*. Auch dieser Begriff ist vage, changiert er doch zwischen dem bloß physischen „Überleben“ (ZK 107) und dem ökonomischen „Erfolg“ (ebd.) bzw. egoistischen Interesse, bezieht sich meist auf Individuen, aber auch auf nationale Kollektive oder die Gattung. Vermutlich handelt es sich um eine Schopenhauer- und/oder Freud-Referenz (analog zur kritischen Wendung von Marquis de Sade in der *Dialektik der Aufklärung*). Schopenhauer geht in seiner Lehre vom *Willen zum Leben* davon aus, dass sich alle Triebe und Bedürfnisse von Lebewesen auf zwei Grundtriebe zurückführen lassen: auf den individuellen Selbsterhaltungstrieb und den übergeordneten Fortpflanzungstrieb, der den Selbsterhalt der Gattung sicherstellt (vgl. Schopenhauer 2018a, 660). Der menschliche Intellekt sei „nur eine höhere Steigerung des thierischen“ (ebd., 165); seinem Ursprung nach ist er ein Instrument oder „Werkzeug“ (ebd., 709) des nach Selbsterhalt strebenden Willens. Der späte Horkheimer hat Schopenhauers Metaphysik denn auch als Vorahnung des Schreckens einer auf technische Rationalität verkürzten Zivilisation gewürdigt (vgl. Horkheimer 1985d). Freud wiederum, der sich mehrfach positiv auf Schopenhauers Willensphilosophie bezog (vgl. u.a. Freud 2000a [1905], 46; Freud 2000c [1932], 540), unterschied in seiner ersten Triebtheorie zwischen dem Realitätsprinzip der Selbsterhaltungs- oder Ich-Triebe und dem Lustprinzip der sublimierbaren Sexualtriebe (vgl. Freud 2000b [1911]). Zumindest bei Adorno ist offensichtlich, dass er bei ‚Selbsterhalt‘ an Freud denkt, umschreibt er doch die „anwachsende Selbsterhaltung“ als „Anwachsen des universalen Ich-Prinzips“ (Adorno 2001, 26). Die sozialphilosophische These ist eine nochmalige Verschärfung der dualistischen Trieblehre Freuds: Die Menschen verengen ihr Leben auf das Realitätsprinzip.^6^

Das Realitätsprinzip ermahnt laut Horkheimer zu Konformität, zu flexibler Anpassung an das, was die Gesellschaft gerade einfordere. Um der Selbsterhaltung willen muss sich das Selbst seiner substantiellen, das heißt flexibilitätshemmenden Bestimmungen entledigen – und dieser „Prozess der Anpassung ist jetzt vorsätzlich und deshalb total geworden“ (ZK 108). Selbsterhaltung ohne Selbst. „Als Endresultat des Prozesses haben wir auf der einen Seite das Selbst, das abstrakte Ich, jeder Substanz entleert bis auf seinen Versuch, alles im Himmel und auf Erden in ein Mittel seiner Erhaltung zu verwandeln; und auf der anderen Seite haben wir eine leere, zu bloßem Material degradierte Natur, bloßen Stoff, der zu beherrschen ist, ohne jeden anderen Zweck als eben den seiner Beherrschung.“ (ZK 109) Am Ende der Aufklärung steht eine Vernunft, die nur noch pragmatische Funktionen erfüllt. Sie hat lediglich noch die passenden Instrumente zu identifizieren – und auch das Selbst so zuzurichten, dass es reibungslos als Werkzeug im herrschenden Getriebe fungieren kann. „Vernunft selbst wird mit diesem Anpassungsvermögen identisch.“ (ZK 109)

Diese Grundthese wirkt merkwürdig lebensfremd – ganz gleich, ob man die Kritik der instrumentellen Vernunft als Kritik einer verabsolutierten Mittelsuche oder als Kritik einer Reduktion des Lebens auf konformistischen Selbsterhalt liest. Denn fragt man die Menschen, was in ihrem Leben Bedeutung hat oder für sie von Wert ist, so werden sie nicht zuletzt *die* Punkte nennen, die Horkheimer zufolge zu bloßen Mitteln degradiert werden: Glück, Gesundheit, Reichtum. Wahrscheinlich werden sie die Liste noch ergänzen durch Ziele wie Freundschaft, Familie oder Selbstverwirklichung. Zwar kann Horkheimer auf gewisse Alltagsphänomene verweisen – wie die Funktionalisierung von Gesundheit und Entspannung für berufliche Zwecke (vgl. ZK 56) –, aber die Tatsache, dass gewisse Zwecke *auch* Mittel für andere Zwecke sind, belegt nicht, dass sie *lediglich* Mittel sind. Gesundheit und Entspannung beispielsweise sind gleichbedeutend mit körperlichem Wohlbefinden – und wer betrachtet sein Wohlbefinden schon unter *rein* funktionalistischen Aspekten? Dass die radikale Funktionalisierungsthese nicht aufgeht, lässt sich selbst noch an der Sirenen-Erzählung der Odyssee aufzeigen. Die Vernunft des Odysseus besteht ja nicht nur darin, die Sirenen zu überlisten (und damit dem sicheren Tode zu entgehen), sondern sie steht zugleich im Dienst des Sinnengenusses: Odysseus will sicher in seine Heimat zurückkehren *und* den Gesang der Sirenen vernehmen. Seine Vernunft ist nicht eindimensional-berechnend, keine bloße Herrschaftstechnik, sondern versucht noch unter den schwierigsten Bedingungen widerstreitenden Ansprüchen gerecht zu werden (vgl. Welsch 1995, 91).

### Die moralphilosophischen Implikationen

Horkheimers Grundthese hat kaum zu unterschätzende Konsequenzen für die Ideologie- (1), Staats- (2) und Moraltheorie (3). Mein Fokus liegt auf den moralphilosophischen Implikationen, weshalb ich auf die Ideologie- und Staatstheorie nur kurz eingehen werde.

1. Die Idee, Vernunft erschöpfe sich weitgehend in einer instrumentellen Rationalität, die den Selbsterhaltungszwecken der Subalternen oder den Interessen verschworener Machtcliquen dient, lässt nur wenig Raum für die Bedeutung, die politische und religiöse Weltanschauungen für die handelnden Akteure haben. In Bezug auf den Nationalsozialismus mündet sie gar in eine Verschwörungs- und Manipulationstheorie (vgl. Schäfer 1994). So gerät der Volksgemeinschaftsgedanke zu einem bloßen „Schleier“, unter dem die vormals konkurrierenden „ökonomischen Gewalten […] eine gemeinsame Gewalt gegen das [deutsche, S.E.] Volk“ (ZK 111) bildeten. „Aber da es einer fortwährenden Propaganda unterworfen war, war das Volk darauf vorbereitet, sich passiv den neuen Machtverhältnissen anzupassen.“ (ebd.) Selbst die „nazistischen Pogrome“ seien „im gegebenen Augenblick befohlen und von oben abgeblasen“ (ZK 130) worden – wobei „von oben“ die „Nazis und ihre industriellen und militärischen Hintermänner“ (ZK 130) meint. Zwar argumentiert Horkheimer, dass der NS-Ideologie auch ein psychologisches Bedürfnis entgegengekommen sei. Die Identifikation mit der „Rasse, dem Vaterland, dem Führer“ (ZK 123) sei eine konformistische Rebellion gegen die Versagungen gewesen, die die „Zivilisation“ (ZK 120) den Menschen auferlegt. Die Herrschenden verstanden es demnach, den aus den Versagungen resultierenden Hass der Bevölkerung bewusst umzulenken. „Die Nazis manipulierten die unterdrückten Wünsche des deutschen Volkes.“ (ZK 130)^7^ Die Entsagungen, die Horkheimer nennt, sind jedoch sehr allgemein gehalten und tragen kaum etwas zum Verständnis der für die NS-Ideologie charakteristischen Projektionen bei. Es ist von den „elterlichen Ermahnungen“ die Rede, „nicht unordentlich zu sein und nicht zu vergessen, sich hinter den Ohren zu waschen“ (ebd.); erwähnt wird auch die Unterdrückung sexueller Wünsche durch die Institution der Ehe (ZK 121) und die Unterdrückung mimetischer Impulse (ZK 124 ff.). Der einzige Punkt, der über allgemeine zivilisatorische Triebhemmungen hinausgeht, ist das, was Alexander Mitscherlich später die ‚vaterlose Gesellschaft‘ nannte: die „zunehmende Übertragung“ der „erzieherischen Funktionen auf die Schule und die sozialen Gruppen“ (ZK 124). Diese Entwicklung wird von Horkheimer insofern als problematisch eingestuft, als sich nun kein grundlegender Konflikt des Kindes mit dem Vater mehr einstelle; dieser Konflikt sei jedoch eine notwendige Durchgangsstufe zur Etablierung psychischer Resilienz. Selbst wenn man diese (umstrittene) These akzeptiert, handelt es sich zunächst nur um einen *Beitrag* zur Erklärung von (Autoritarismus ermöglichender) *Ich-Schwäche*. Abgesehen davon, dass es noch weitere (und gewichtigere) Faktoren gibt, die eine Schwächung des Ichs hervorrufen, trägt die These nichts zur Erklärung genuin moderner antisemitischer Projektionen bei.^8^

2. Im Vergleich zu avancierten marxistischen Theorien verändert sich das Verständnis des Staates bei Horkheimer fundamental. Während sich im Anschluss an Eugen Paschukanis (1969) der moderne Staat insofern als Staat des Kapitals begreifen lässt, als er sein Gewaltmonopol dafür einsetzt, die Aneignungsnormen kapitalistischer Marktvergesellschaftung auf neutrale Art und Weise abzusichern, leitet Horkheimer aus vermeintlichen ökonomischen Gesetzmäßigkeiten einen Übergang von der anonymen Herrschaft des Marktes zur personalen Herrschaft der Rackets ab. In Kurzform: Die Konkurrenz des Marktes führt unweigerlich zu Konzentrationsprozessen, aus denen ein krisengeschüttelter Monopolkapitalismus hervorgeht, der wiederum, um den Status quo der Herrschaft zu stabilisieren, durch eine staatliche Befehlswirtschaft abgelöst wird. In dieser sind nicht länger Privateigentum und Profit ausschlaggebend für die gesellschaftliche Stellung und die Verteilung der Produktionsfaktoren, sondern politische Macht: Weltweit setzt sich der Staatskapitalismus (Pollock 1981) durch, sei es in einer liberalen (New Deal) oder autoritären Variante (Nationalsozialismus, Sowjetunion). Zwar werde der Profit im Staatskapitalismus weiterhin privat angeeignet, seine Lenkungsfunktion büße der Markt jedoch ein: „Das Dorado der bürgerlichen Existenzen, die Sphäre der Zirkulation, wird liquidiert“ (Horkheimer 1987, 293). Wie Franz Neumann in seiner Studie Behemoth (1998) durch eine Fülle empirischen Materials belegte, traf die These, die kapitalistischen Produktionsverhältnisse seien durch eine gänzlich neue Form der (Staats‑)Ökonomie ersetzt worden, jedoch noch nicht einmal für das nationalsozialistische Deutschland zu (siehe hierzu Hirsch 2014). Selbst wenn man die Staatskapitalismusthese im Hinblick auf Länder wie China oder Indien als einen instruktiven Beitrag zur Erforschung der Varieties of Capitalism liest (Ten Brink/Nölke 2013), so lässt sich doch festhalten, dass Horkheimer und Pollock deutlich mehr im Sinn hatten: Vor dem Hintergrund kulturanthropologischer Erwägungen und traditionsmarxistischer Prämissen gingen sie von einem zielgerichteten Prozess aus, der von ihnen jedoch weder theoretisch plausibel begründet war noch sich geschichtlich bewahrheitet hat. Die Zirkulationssphäre ist nicht liquidiert. In der OECD-Welt gibt es zwar immer wieder besondere Situationen, in denen sich der Staat gezwungen sieht, das Wirtschaftsgeschehen mittels Investitions‑, Steuer- und Währungspolitik in stärkerem Maße zu prägen (wie momentan in der Covid-19-Pandemie), jedoch sind diese Eingriffe erstens nicht von Dauer und sie dienen zweitens der Stabilisierung privatkapitalistischer Akkumulation. Staatskapitalismus, zu Ende gedacht, ist ein Widerspruch in sich (vgl. Brick/Postone 1982, 189 ff.).

3. Das Projekt einer Kritik der instrumentellen Vernunft folgt der Idee, die Aufklärung über sich selbst aufzuklären. Sie soll die innere Logik und die Konsequenzen einer Vernunft offenlegen, die der alles erfassenden naturbeherrschenden Rationalität verpflichtet ist. Habermas zufolge beruht das Projekt auf einem performativen Widerspruch: Horkheimer und Adorno beschreiben „die Selbstzerstörung des kritischen Vermögens auf paradoxe Weise, weil sie im Augenblick der Beschreibung noch von der totgesagten Kritik Gebrauch machen“ (Habermas 1985, 144). Dabei kann sich Habermas auf gewisse Aussagen in der *Dialektik der Aufklärung* stützen. So heißt es in dem auf Horkheimer zurückgehenden Juliette-Exkurs, „die etablierte bürgerliche Ordnung“ habe „Vernunft *vollends* funktionalisiert.“ (DdA 108, Herv. von mir) Vernunft „ist zur zwecklosen Zweckmäßigkeit geworden, die eben deshalb sich in alle Zwecke spannen läßt. Sie ist der Plan an sich betrachtet.“ (ebd.) Meistenteils ist Horkheimer bei seiner Wortwahl jedoch vorsichtiger. In *Zur Kritik der instrumentellen Vernunft *schreibt er beispielweise: „Vernunft selbst *wird* mit diesem Anpassungsvermögen identisch“ (ZK 109, Herv. von mir). Dies ist eine Entwicklungs- oder Tendenzaussage. Zwar beinhaltete sie, dass sich Vernunft immer weiter an instrumenteller Vernunft assimiliert, jedoch behauptet Horkheimer nicht, dass dieser Prozess des Identischwerdens bereits abgeschlossen ist – und nur wenn er abgeschlossen wäre, handelte es sich um einen performativen Widerspruch. Gleichwohl zeigt Habermas’ Kritik in die richtige Richtung – und zwar insofern, als die *normative Quelle und die normativen Anknüpfungspunkte *der geübten Kritik seltsam unterbelichtet und vage bleiben. Die wenigen diesbezüglichen Stellen haben bei Horkheimer entweder kognitivistische Anklänge – z.B. wenn er sich die Versöhnung mit der Natur davon erhofft, „ihr scheinbares Gegenteil zu entfesseln, das unabhängige Denken“ (ZK 135) – oder sie rekurrieren auf eine emotionale moralische Disposition: „die Menschen sind gewöhnlich viel besser als das, was sie sagen, denken oder tun“ (ZK 157). In welcher Hinsicht sind sie besser? Wenn Menschen besser sind als ihre Äußerungen, Gedanken und Handlungen, bleiben noch ihre *Gefühle* übrig. Tatsächlich hat nicht nur Adorno (1997, 358), sondern bereits der frühe Horkheimer argumentiert, es gebe einen somatischen normativen Impuls, der sich als Reaktion auf die Wahrnehmung von Leid einstellt. In *Materialismus und Moral* von 1933 spricht er in Anlehnung an Kant von einem „moralische[n] Gefühl“ (Horkheimer 1988, 133), das er jedoch nicht, wie Kant, als eine Wirkung reiner praktischer Vernunft versteht. Er beschreibt dieses Gefühl als eine Form von Liebe, die sich unter den gegenwärtigen Verhältnissen als Mitleid (ebd., 136) und emanzipatorische Politik (ebd., 137) artikuliert. Diese Liebe wünscht allen Menschen „die freie Entwicklung ihrer fruchtbaren Kräfte. Es scheint ihr, als hätten die lebenden Wesen einen Anspruch auf Glück, und sie fragt nicht im geringsten nach einer Rechtfertigung oder Begründung dafür.“ (ebd., 134) Dass sich hinter Horkheimers Aussage, „der Mensch“ sei „immer noch besser [...] als die Welt, in der er lebt“ (ZK 162), die anthropologische Überzeugung einer normativen, selbst unter maximalem Sozialisationsdruck nicht verstummenden Affektivität verbirgt, ist auch insofern naheliegend, als Horkheimer sich stets der Philosophie Arthur Schopenhauers verpflichtet fühlte. Schopenhauer zufolge hat eine Handlung nur dann moralischen Wert, wenn sie der emotionalen Teilnahme am Leid anderer entspringt:

Wenn nun aber meine Handlung ganz allein *des Andern wegen* geschehen soll; so muß *sein Wohl und Wehe unmittelbar mein Motiv* sein: so wie bei allen andern Handlungen das *meinige* es ist. Dies bringt unser Problem auf einen engern Ausdruck, nämlich diesen: wie ist es irgend möglich, daß das Wohl und Wehe *eines Andern*, unmittelbar, d.h. ganz so wie sonst nur mein eigenes, meinen Willen bewege, also direkt mein Motiv werde, und sogar es bisweilen in dem Grade werde, daß ich demselben mein eigenes Wohl und Wehe, diese sonst alleinige Quelle meiner Motive, mehr oder weniger nachsetze? – Offenbar nur dadurch, daß jener Andere *der letzte Zweck* meines Willens wird, ganz so wie sonst ich selbst es bin: also dadurch, daß ich ganz unmittelbar *sein* Wohl will und *sein* Wehe nicht will, so unmittelbar, wie sonst nur das *meinige*. Dies aber setzt nothwendig voraus, daß ich bei *seinem* Wehe als solchem geradezu mitleide, *sein* Wehe fühle, wie sonst nur meines, und deshalb sein Wohl unmittelbar will, wie sonst nur meines. Dies erfordert aber, daß ich auf irgend eine Weise *mit ihm identificirt* sei, d.h. daß jener gänzliche *Unterschied* zwischen mir und jedem Andern, auf welchem gerade mein Egoismus beruht, wenigstens in einem gewissen Grade aufgehoben sei. (Schopenhauer 2018b, 564)

Horkheimer hat nicht nur die Relevanz Schopenhauers für die Herausbildung der Kritischen Theorie hervorgehoben – „Die beiden Philosophen, welche die Anfänge der Kritischen Theorie entscheidend beeinflußt haben, waren Schopenhauer und Marx“ (Horkheimer 1985e, 336) –, sondern auch explizit seine Ethik gewürdigt: „Im Gegensatz zur heutigen Gesinnung bietet seine Metaphysik die tiefste Begründung der Moral“ (Horkheimer 1985d, 515 f.).

Bis hierhin lässt sich festhalten, dass es für eine Kritik der gegenwärtigen Gesellschaft des Zusammenspiels von Reflexion (Entfesselung des unabhängigen Denkens) und Affektivität bedarf. Nur welche Rolle kommt der Reflexion genau zu? Diese Frage ist alles andere als leicht zu beantworten, denn Horkheimers Ausführungen zur Vernunft lassen mindestens zwei verschiedene Interpretationen zu.

*Erstens*: Es finden sich Formulierungen, in denen Vernunft auf ein herrschaftsdienliches Vermögen der Abstraktion, Subsumtion und Kalkulation *reduziert* wird. In *Zur Kritik der instrumentellen Vernunft* unterscheidet Horkheimer zwar zwischen subjektiver und objektiver Vernunft – das ganze 1. Kapitel ist dieser Unterscheidung gewidmet –, jedoch finden sich auch Stellen wie diese: „Die Vernunft kommt zu sich selbst, indem sie […] sich als bloßes Instrument versteht.“ (ZK 21) Analog spricht er im Juliette-Exkurs von der „reinen Vernunft“ als einer „inhaltslosen Verfahrungsweise“ (DdA 110). Die Vernunft *als solche* ist ein formales Vermögen der Mittelwahl und kann folglich keine eigenständige Rolle bei der moralischen Zwecksetzung spielen: „Die Unmöglichkeit, aus der Vernunft ein grundsätzliches Argument gegen den Mord vorzubringen, nicht vertuscht, sondern in alle Welt geschrieen zu haben, hat den Haß entzündet, mit dem gerade die Progressiven Sade und Nietzsche heute noch verfolgen.“ (DdA 140) Man ist geneigt, diese und andere reduktionistische Formulierungen für eine bloß abkürzende Redeweise zu halten. Wenn bei Horkheimer von *der* Vernunft die Rede ist, sei eigentlich nur ihre (nun alles dominierende) *subjektive* Seite gemeint. Tatsächlich lassen sich einige Formulierungen, die auf den ersten Blick reduktionistisch klingen, durch die Berücksichtigung ihres Kontextes in diesem Sinne aufklären. Der Rettungsversuch gelingt jedoch nicht immer. Neben der Ambiguität der verwendeten Termini (wie Vernunft, Aufklärung, Herrschaft etc.) gibt es nämlich auch sachliche Gründe für bestimmte reduktionistische Formulierungen. In *Materialismus und Moral* ist Horkheimers moral*soziologisches* Motiv ausschlaggebend, das Phänomen der Moral auf das für die kapitalistische Gesellschaft konstitutive Auseinanderfallen von individuellem und allgemeinem Interesse zurückzuführen; der kategorische Imperativ sei nicht Ausdruck reiner praktischer Vernunft, sondern historisch-spezifischer Produktionsverhältnisse.^9^ Horkheimer argumentiert hier offen gegen den ethischen Universalismus und Rationalismus: „Der Materialismus vermutet hinter der Moral keine überhistorische Instanz.“ (Horkheimer 1988, 131) „Verbindliche moralische Gebote existieren nicht.“ (ebd., 133) Moral „ist keiner Begründung fähig – weder durch Intuition noch durch Argumente.“ (ebd.) Kritische Theorie macht es sich folglich zur Aufgabe, das aus dem Leiden und Mitleiden entstehende Interesse an sozialer Veränderung zu artikulieren. Und auch in der *Dialektik der Aufklärung* sind Formulierungen, die Vernunft mit formaler Rationalität gleichsetzen, kein Zufall. Der sachliche Grund ist die entwicklungsgeschichtliche Fundierung des Vernunftvermögens in dem menschlichen Bestreben, über die äußere Natur zu gebieten (ein Bestreben, das bereits den frühen magischen Ritualen zugrunde gelegen habe): Das begriffliche Denken sei entstanden als Funktion der Selbsterhaltung, beruhe grundlegend auf Repression. Ausgehend von dieser materialistisch-genetischen Perspektive ist der Siegeszug formaler Rationalität nur folgerichtig: Vernunft kommt „zu sich selbst“ (ZK 21). Wenn dem aber so ist, wie kann sie dann noch zur Kritik der bestehenden Weltbeziehungen beitragen? Die Antwort der *Dialektik der Aufklärung* lautet *Selbstreflexion der Vernunft*. Mit Autoren wie Sade und Nietzsche gilt es „die Aufklärung sich über sich selbst entsetzen zu lassen“ (DdA 139). Warum sollte sie sich aber entsetzen? Wenn Vernunft als zweckindifferente, emotionsabstinente und darum universell applizierbare Vernunft zu sich selbst gekommen ist (ihrem Begriff entspricht), wie soll sie sich dann qua Selbstreflexion zu ethischen Zielen wie Solidarität, Liebe und Glück verpflichten können (vgl. Schnädelbach 1986, 71)? Wie soll sich eine ihres Formalismus innewerdende Vernunft transzendieren können, wenn sie nicht bereits das Vermögen moralischer Zwecksetzung in sich trägt? Entweder trägt sie das Vermögen in sich – dann würde man inhaltlich gerne mehr darüber erfahren und wünschte sich eine präzisere Terminologie. Oder sie trägt es *nicht* in sich. Dann kann den entscheidenden Anstoß wohl nur noch die somatische Reaktion im Anblick des alltäglichen Leids geben. „Einem jeden sind Situationen vertraut, die ihrem ganzen Wesen nach […] eine bestimmte Richtlinie des Handelns vorschreiben – zum Beispiel ein Kind oder ein Tier in der Gefahr des Ertrinkens, eine hungernde Bevölkerung oder eine individuelle Krankheit. Jede dieser Situationen spricht sozusagen seine eigene Sprache.“ (ZK 34) Objektive Vernunft – um sie geht es im Kontext dieses Zitats – vernimmt diese situationsspezifische Sprache des Leids und reagiert ihr entsprechend, setzt also das *unabhängig von ihr* Gebotene in Handlung um. Unter objektiver Vernunft versteht Horkheimer nämlich zweierlei: Zum einen „die der Wirklichkeit innewohnende Struktur“, zum anderen soll der Begriff die „Fähigkeit kennzeichnen, eine solche objektive Ordnung zu reflektieren.“ (ZK 34) Objektive Vernunft ist weniger eine Zweck*setzungs*- denn eine Zweck*befolgungskompetenz*. Nach kantischen Maßstäben ist sie damit ebenso passiv und heteronom wie die subjektive Vernunft des Selbstinteresses. Darüber hinaus stellt sich die Frage, aus welchem Grund sich die (ihrem Begriff nach formale) Vernunft für Imperative empfänglich zeigen sollte, die dem Leid und Mitleid entspringen. Wie Horkheimer im Juliette-Exkurs über mehrere Seiten ausführt (DdA 121 ff.), ist eher das Gegenteil zu erwarten: Die herrschaftsaffine Vernunft verfolgt das Mitleid erbarmungslos als lästige, ihr im Weg stehende Sünde.

*Zweitens*: Diesem ersten Vernunft-Verständnis direkt entgegengesetzt muten Aussagen an, denen zufolge die derzeit dominierende technische Vernunft nur die zur Unkenntlichkeit entstellte Variante einer Vernunft ist, die *eigentlich* auf humanistische Zwecke gerichtet ist. So wird die instrumentelle Vernunft als „Unvernunft“ (DdA 110), also als das *Gegenteil* von Vernunft bezeichnet. Gleich mehrere Formulierungen gehen in eine ähnliche Richtung. Es ist von „der Erniedrigung der Vernunft“ (ZK 71), der „Perversion ihrer selbst“ (DdA 113) und „der sich entfremdeten Vernunft“ (DdA 111) die Rede. Wenn Szientismus, Kulturindustrie und autoritärer Staat die Vernunft *erniedrigen* bzw. sie sich als zwecklose Zweckmäßigkeit selbst *fremd* geworden ist, muss sie offenbar ein bestimmtes, ihr entsprechendes normatives Ziel anvisieren, um wieder mit sich selbst ins Reine zu kommen. Hierzu passt, dass Horkheimer zwischen Vernunft (im emphatisch-praktischen Sinne) und Rationalität unterscheidet: „Jene Utopie aber, die zwischen Natur und Selbst die Versöhnung ankündigte, trat […] als Idee des Vereins freier Menschen hervor und zog alle Wut der Ratio auf sich.“ Die Utopie, die dem Herrschaftsprinzip eine Absage erteilt, sei „irrational und vernünftig zugleich“ (DdA 110) gewesen. An Stellen wie diesen wird Kritik scheinbar im Namen einer Vernunft geübt, die sich erst dadurch *gewinnt* oder *zu sich selbst kommt*, dass sie innehält, um ihre eigene Geschichte und ihre verspielten Potentiale zu reflektieren. Die Kritik der verkürzten Vernunft setzt also einen unverkürzten Vernunftbegriff voraus, d.h. ein Vermögen *praktischer Autonomie*. Zu Beginn des Juliette-Kapitels stellt Horkheimer denn auch heraus, dass Kants Begriffe „doppelsinnig“ (DdA 111) sind. Die synthetische Einheit der Apperzeption als oberstes Prinzip des Denkens und Ausgangspunkt aller Erkenntnis sei einerseits „das Produkt sowohl wie die Bedingung der materiellen Existenz“ (DdA 106), andererseits enthalte das reine Ich „die Idee eines freien Zusammenlebens der Menschen, in dem sie zum allgemeinen Subjekt sich organisieren“ (DdA 102). Die Wendung, dass sich im abstrakten, von allen inhaltlichen Bestimmungen absehenden Ich das Freiheits- und Gleichheitsbewusstsein Bahn bricht, erinnert an Hegel: „Es gehört der Bildung, dem *Denken* als Bewußtseyn des Einzelnen in Form der Allgemeinheit, daß *Ich* als *allgemeine* Person aufgefaßt werde, worin *Alle* identisch sind. Der *Mensch gilt so, weil er Mensch ist*, nicht weil er Jude, Katholik, Protestant, Deutscher, Italiener u.s.f. ist.“ (Hegel 2009, 175, § 209 A) Die Parallelen zu Hegel sind auch ansonsten verblüffend, beschreibt er doch eine ähnliche Dialektik wie Horkheimer: Die Fähigkeit, mittels der Abstraktion den Begriff des reinen Ichs zu bilden, bildet zwar die Grundlage der Anerkennung von jedermann als Rechtsperson, jedoch könne Freiheit nicht auf ihre negative Seite reduziert werden, ohne einen pathologischen Prozess in Gang zu setzen, der die Freiheitsgewinne wieder kassiert. Freiheit, die jedes inhaltliche Moment von sich fernhält, tilgt in ihrem krampfhaften Bemühen um Selbstbehauptung auch jede Bedeutung, jeden Sinn, jede Bindung. Auf der Ebene des Einzelnen mündet sie in Handlungsunfähigkeit, Dezisionismus oder Eitelkeit, auf gesellschaftlicher Ebene im fanatischen Kampf gegen alle gewachsenen Institutionen: Qualitätslos, wie er ist, hat der negativ bestimmte Wille nur als „Furie des Zerstörens“ (Hegel 2009, 33, § 5 A) noch ein „Gefühl seines Daseyns“ (ebd., 32 f., § 5 A). Die Aufklärungskritik der Kritischen Theorie liest sich ganz ähnlich: „Allen Stoff empfängt sie [die Aufklärung, S.E.] von den Mythen, um sie zu zerstören.“ (DdA 28). Die neuzeitliche Variante der Aufklärung hat mit besonderer „Radikalität“ (DdA 111) die mythischen Weltbilder destruiert, d.h. den Glauben an alles, das fraglos zu gelten beansprucht, erschüttert und durch pragmatisches Regel- und Wahrscheinlichkeitswissen ersetzt. Als soziale Folge dieser Entwicklung wurde mit den Autoritäten des Ancien Régime kurzer Prozess gemacht; die bürgerliche Gesellschaft trat an seine Stelle. Das Destruktionswerk der Aufklärung war damit jedoch nicht beendet. Fortan arbeitete sie daran, die geistigen Fundamente der bürgerlichen Gesellschaft zu untergraben, als naiven Glauben zu denunzieren:

Alle vorgegebenen Bindungen verfielen […] dem tabuierenden Verdikt, nicht ausgenommen solche, die zur Existenz der bürgerlichen Ordnung selbst notwendig waren. Das Instrument, mit dem das Bürgertum zur Macht gekommen war, Entfesselung der Kräfte, allgemeine Freiheit, Selbstbestimmung, kurz, die Aufklärung, wandte sich gegen das Bürgertum […]. Aufklärung macht ihrem Prinzip nach selbst vor dem Minimum an Glauben nicht halt, ohne das die bürgerliche Welt nicht existieren kann. […] Ihre anti-autoritäre Tendenz, die, freilich bloß unterirdisch, mit jener Utopie im Vernunftbegriff kommuniziert, macht sie dem etablierten Bürgertum schließlich so feindlich wie der Aristokratie […]. Das anti-autoritäre Prinzip muß schließlich ins eigene Gegenteil, in die Instanz gegen die Vernunft selber umschlagen: die Abschaffung alles von sich aus Verbindlichen, die es leistet, erlaubt es der Herrschaft, die ihr jeweils adäquaten Bindungen souverän zu diskreditieren und zu manipulieren. (DdA 112 f.)

Horkheimer lässt die Strenge der begrifflichen Differenzierung, die man von Hegel gewohnt ist, hier zwar vermissen, aber es ist offensichtlich, dass seine Überlegungen in eine vergleichbare Richtung gehen. Wenn das Bürgertum das Instrument der *Freiheit und Selbstbestimmung* genutzt hat, um an die Macht zu gelangen, dieses Instrument seiner inneren Natur nach jedoch *anti-autoritär* ist, weshalb es im weiteren Geschichtsverlauf selbst noch die verbliebenen Grundüberzeugungen (des Bürgertums) angriff und die im Vernunftbegriff angelegte Utopie vereitelte, beschreibt Horkheimer die selbstzerstörerische Konsequenz einer Freiheit, die eine ihrer beiden Seiten – die negative Seite, das Moment der *reinen Unbestimmtheit* (Hegel 2009, 32, § 5) – zum Absoluten erklärt und damit jeden Inhalt aus sich ausschließt. Wie Hegel kritisiert Horkheimer den kategorischen Imperativ denn auch als *formalistisch*.

Wenn die Parallelen aber so offensichtlich sind, was hindert Horkheimer dann daran, offensiv hegelianisch zu argumentieren? Wenn schon Hegel eine vergleichbare Dialektik der Aufklärung geschrieben hat, wie erklärt sich dann die offensichtliche Diskrepanz zur hegelschen Vernunftemphase? Ich denke, dass hier zwei Gründe ausschlaggebend waren.

Der erste Grund hängt mit einer Hegel-Kritik zusammen, die ich in bestimmter Hinsicht für überzeugend halte. In seiner Rechtsphilosophie begnügt sich Hegel nicht mit der unproblematischen Feststellung, dass mit den individualisierenden Effekten der negativen Freiheit erst die notwendigen Bedingungen für höhere Formen der Sittlichkeit geschaffen werden. Er versucht darüber hinaus zu zeigen, dass die negative Freiheit, die vor allem durch die bürgerliche Gesellschaft forciert wird, schon *von sich aus* zur Etablierung sittlicher Institutionen führt. Die negative Freiheit soll notwendige *und* hinreichende Bedingung ihrer Selbsttranszendierung sein. Ein Beispiel hierfür sind die das Profitstreben limitierenden Berufsgenossenschaften (die Korporationen), die Hegel noch aus dem Profitstreben selbst hervorgehen lässt. Nun kennt auch Horkheimer positive nicht-intendierte Effekte: Er entlarvt zwar zu Beginn des Juliette-Exkurses die Erkenntnistheorie als Ausdruck des Herrschaftsstrebens – „Selbsterhaltung ist […] die Seele der Kategorientafel“ (DdA 106) –, sieht in der transzendentalen Einheit der Apperzeption jedoch ein nicht-intendiertes Emanzipationspotential. Der Unterschied zu Hegel: Die Radikalisierung instrumentellen Denkens verhindert, dass dieses Potential ausgeschöpft wird; instrumentelles Denken führt nicht über sich selbst hinaus, sondern nur tiefer ins Verhängnis hinein.

Der zweite Grund, der Horkheimer daran hindert, die hegelsche Vernunftemphase zu übernehmen, ist seine Tendenz, die Selbst- und Fremdbezüge der Selbsterhaltung mit Herrschaftsverhältnissen zu identifizieren. Schon im ersten Satz der *Dialektik der Aufklärung* wird beides zusammengedacht: „Seit je hat Aufklärung […] das Ziel verfolgt, von den Menschen die Furcht zu nehmen und sie als Herren einzusetzen.“ (DdA 19) Dass hier ein Herrschaftsbegriff im engeren Sinne vorliegt, wird im Verlauf der Argumentation überdeutlich: „Die Aufklärung verhält sich zu den Dingen wie der Diktator zu den Menschen.“ (DdA 25) Die Konsequenzen liegen auf der Hand. Wenn Selbsterhaltung und Herrschaft bisher zusammenfielen und wenn sich das Ich des Menschen und sein Denkapparat freudianisch auf Selbsterhaltung zurückführen lassen, dann ist der Vernunft – Potential hin oder her – von Grund auf zu misstrauen. Während bei Hegel die Verwirklichung der Vernunft durch eine List verbürgt ist – die Vernunft realisiert ihre objektiv-sittlichen Zwecke vermittelt über subjektiv-selbstsüchtige Zwecke –, perpetuiert die Vernunft nach Horkheimer Herrschaftsverhältnisse, weil diese von jeher ihr Zweck waren: Entwickelt sich die Vernunft doch erst durch den Gegensatz zur „Natur, das Substratum nie endender Subsumtion in der Idee, nie endender Unterwerfung in der Wirklichkeit“ (DdA 132) – und diese „Herrschaft über die Natur reproduziert sich innerhalb der Menschheit.“ (DdA 130) Die Lage scheint hoffnungslos zu sein. Einerseits sind sich Horkheimer und Adorno bewusst, dass „die Freiheit in der Gesellschaft vom aufklärenden Denken unabtrennbar ist“, andererseits – „und darin liegt unsere petitio principii“ (DdA 13) – sind Vernunft und Aufklärung aufs Innigste verbunden mit Repression und Gewalt. Die Entwicklung, die die Zivilisation genommen hat, ist kein Zufall; die Menschen hatten nie eine wirkliche Wahl: „Das Wesen der Aufklärung ist die Alternative, deren *Unausweichlichkeit* die der Herrschaft ist. Die Menschen hatten immer zu wählen zwischen ihrer Unterwerfung unter Natur oder der Natur unter das Selbst.“ (DdA 49, Herv. von mir) Dass die Situation darum aussichtlos ist, mochte sich Horkheimer freilich nicht eingestehen. Stattdessen greift er zu der Idee, auf ihrem Höhepunkt könne die Katastrophe doch noch in Freiheit und Humanität umschlagen: „Die industrielle Disziplin, der technische Fortschritt und die wissenschaftliche Aufklärung, gerade die ökonomischen und kulturellen Prozesse, die die Auslöschung der Individualität bewirken, versprechen – obgleich die Anzeichen gegenwärtig schwach genug sind – ein neues Zeitalter einzuleiten, in dem die Individualität als Element in einer weniger ideologischen und humaneren Daseinsform neu entstehen kann.“ (ZK 163) Warum ausgerechnet die Prozesse, die die Individualität auslöschen, ein Zeitalter der Individualität einleiten sollen, wird leider nicht erläutert. Die Argumentationsfigur erinnert an die Revolutionstheorie des frühen Marx, für den das universelle Leiden des Proletariats die Befreiung der Menschheit verbürgte. Sie erinnert auch an Hegels Begriff der bestimmten Negation. Aber welche ideengeschichtlichen Bezüge man auch immer herstellen mag, der Gedanke, dass die Auslöschung von Freiheit zu Freiheit führe, ist ohne Angabe konkreter sozialer Faktoren schlicht nicht nachvollziehbar. Lässt man den Gedanken fallen, bleibt nur der resignative Befund übrig. Der wäre zwar schlimm für alle sozialen Bewegungen, die sich emanzipativer Ideen verschrieben haben, aber möglicherweise ist er ja zutreffend; kritische Theorie hat, so könnte der Einwand lauten, nicht Hoffnung zu verbreiten, sondern nüchtern zu sagen, was ist. Wie ist es also um die Hauptthesen bestellt?

### Einseitigkeiten der Verfallsgeschichte

Sie sind vor allem einseitig. Die als Urgeschichte der menschlichen Subjektivität präsentierte Kritik der instrumentellen Vernunft ist eine Konstruktion, die letztlich genau das vollzieht, was sie ihrem Gegenstand vorwirft: „sie schneidet das Inkommensurable weg“ (DdA 29). Und zwar in mehrfacher Hinsicht:

1. Habermas kritisiert v.a. das Moderne-Verständnis von Horkheimer und Adorno. Die Moderne zeichnet sich Habermas zufolge durch eine „Ausdifferenzierung der Wertsphären“ (Habermas 1985, 137) aus, deren Entlarvung als *bloßer* Schein herrschaftsförmiger Selbsterhaltung nicht überzeugend ist. Vielmehr ermöglicht die Wertsphärendifferenzierung es der Vernunft, Fragen der propositionalen Wahrheit (Wissenschaft), der normativen Richtigkeit (Moral) und der Authentizität/des Geschmacks (Ästhetik) gemäß ihrer jeweiligen Eigenlogik zu beantworten. Zwar übe die kapitalistische Wirtschaft unbestreitbar einen starken Rationalisierungsdruck auf die drei Wertsphären aus, aber es könne kaum bestritten werden, dass (a) Wissenschaften nicht *nur* verwertbares Wissen produzieren, (b) universalistische Überzeugungen von Recht und Moral „in den Institutionen der Verfassungsstaaten, in Formen der demokratischen Willensbildung, in individualistischen Mustern der Identitätsbildung *auch* eine (wie immer verzerrte und unvollkommene) Verkörperung gefunden haben“ (ebd. 138) und (c) die moderne Kunst nicht in Kulturindustrie aufgeht, sondern in einem gewissen Rahmen konventionalitätsentlastete und nicht auf ökonomische Zwecke festgelegte Subjektivität freisetzt. Wer es mit der älteren Kritischen Theorie hält, mag nun einwenden, dass sie weniger einseitig gewesen ist, als Habermas es ihr unterstellt. In *Art und Mass Culture*, erschienen in der letzten Ausgabe der *Zeitschrift für Sozialforschung*, schildert Horkheimer nicht nur, wie die manipulative Massenkultur die Widerstandskraft der individuellen Erfahrung aufzehrt, er sieht auch ein gewisses „Zeichen der Hoffnung“ in den „grotesken, dissonantischen Ausdruckformen“; in der „Prosa von Joyce etwa und in Bildern wie Picassos *Guernica*“ (Horkheimer 1988b, 424) gibt Kunst „den geschändeten Menschen ein schockierendes Bewusstsein ihrer verzweifelten Situation“ (ebd., 427). Ist der Streit also bloß gradueller Natur? Berücksichtigt man die geschichtsphilosophische Klammer, die Aufsatz, Exkurse und Anhänge der *Dialektik der Aufklärung* sowie die um dieses Buch herum entstandenen Schriften zusammenhält, kann die Frage nur mit *nein* beantwortet werden. Um die nationalsozialistischen Verbrechen, den stalinistischen Terror und individualitätsfeindliche Tendenzen in den Vereinigten Staaten begreiflich zu machen, entwickeln Horkheimer und Adorno die Idee, die Katastrophen der Moderne seien Folge einer Menschheitsgeschichte, die sich ihnen als Prozess fortschreitender Verdinglichung darstellt. Die Moderne enthält nicht nur totalitäre Potentiale, sondern sie *ist* totalitär, weil das sich in ihr realisierende Prinzip es ebenfalls ist: „Aufklärung ist totalitär“ (DdA 41).

2. Durch die gattungsgeschichtliche Rahmung der Kritik entsteht unweigerlich der Eindruck, dass das Verhältnis des Menschen zur Natur der entscheidende Grund für das Dominantwerden der technisch-instrumentellen Rationalität ist. Zumindest für die bürgerlich-kapitalistische Gesellschaft werden damit jedoch Ursache und Wirkung verwechselt. Es sind nicht vorgängige Naturverhältnisse des Menschen, die instrumentelles Denken und soziale Herrschaft induzieren, sondern es sind die sozialformationsspezifischen Verhältnisse der Menschen untereinander, die eine Logik der systematischen Instrumentalisierung von Natur und Geist in Gang setzen. Die große Geschichte, die Horkheimer und Adorno erzählen, stellt die Sache zwangsläufig auf den Kopf. Vor dem Hintergrund der katastrophischen Ereignisse und intellektuellen Regression gelangen sie zu der Überzeugung, den als zu eng empfundenen Horizont der Kapitalismuskritik zu erweitern durch eine Reflexion auf die Grundlagen misslingender Zivilisation insgesamt; sie projizieren das, was sie als gemeinsamen Nenner des Zivilisationsverfalls ausgemacht haben (instrumentelle Rationalität) in die Anfänge der um Selbsterhalt kämpfenden Menschheit, weshalb nun die kapitalistische Warenproduktion und die auf ihr folgenden Totalitarismen der Moderne als Stufen einer Zivilisationsgeschichte erscheinen, die von Anfang an aufs falsche Gleis gesetzt war. Zwar ist es richtig, dass sich bei Horkheimer auch Passagen finden, die den Einfluss der historisch-spezifischen Produktionsverhältnisse betonen (siehe Fußnote 3), jedoch bleiben sie „für das geschichtsphilosophische Zentralargument, das Adorno und Horkheimer ausarbeiten, sekundär.“ (Honneth 1988, 50)

3. Der universalgeschichtliche Ansatz verleitet nicht nur dazu, die Ursache (konkurrenzinduzierte Steigerungslogik des Kapitals) mit der Wirkung (instrumentelle Rationalität) zu verwechseln, sondern auch den Unterschied zwischen bürgerlichen und vorbürgerlichen Gesellschaften unkenntlich zu machen (vgl. Breuer 2016, 72 f.). Genau davor hatte Marx gewarnt. Ihm zufolge bedarf die Rekonstruktion des Werdens eines Gegenstandes zweierlei: Einer genauen Kenntnis des Gewordenen und eines Gespürs für historische Unterschiede. „Die Anatomie des Menschen ist ein Schlüssel zur Anatomie des Affen. Die Andeutungen auf Höhres in den untergeordneten Tierarten können dagegen nur verstanden werden, wenn das Höhere selbst schon bekannt ist. Die bürgerliche Ökonomie liefert so den Schlüssel zur antiken etc. Keineswegs aber in der Art der Ökonomen, die alle historischen Unterschiede verwischen und in allen Gesellschaftsformen die bürgerlichen sehen.“ (Marx 1990, 636) Anachronismen können entweder der Rechtfertigung oder, wie bei Horkheimer und Adorno, der Kritik des Status quo dienen – nichtsdestotrotz bleiben sie Anachronismen. Sie finden sich sowohl in der ersten Abhandlung der *Dialektik der Aufklärung* als auch in den beiden Exkursen. Im Juliette-Kapitel heißt es etwa: „Der Bürger in den sukzessiven Gestalten des Sklavenhalters, freien Unternehmers, Administrators, ist das logische Subjekt der Aufklärung.“ (DdA 102) Der Bürger, dessen „Urbild“ (DdA 61) Odysseus sei, tritt in unterschiedlichen Epochen in unterschiedlichen Gestalten auf, aber jedes Mal handelt es sich um den Souverän, der die äußere und innere Natur malträtiert und über andere Menschen gebietet, als seien sie Gegenstände. Die Kontinuität in der bisherigen Geschichte besteht demnach nicht nur, wie bei Marx, in der Aneignung fremder Arbeit, sondern in der Personalität der Herrschaft. Die Eigenlogik und Eigendynamik der kapitalistischen Strukturen gerät bei Horkheimer und Adorno zur kurzzeitigen Indisponiertheit der Herrschenden – wie das Werkzeug, das sich für einen kurzen Moment selbstständig macht, bevor der Arbeiter es wieder unter seine Kontrolle bringt. Als geistige Nachfahren der Sklavenhalter haben die Machtgruppen des autoritären Staats – die Lenker der faschistischen Massen, die Gewerkschaftsführer und Konzernchefs, die Bürokraten und Militärs – die Zügel wieder fest in der Hand.

4. Grundlage der Anachronismen ist die Annahme, die Unterjochung der äußeren und inneren Natur sei für die Menschen unausweichlich gewesen. „Die Menschen hatten immer zu wählen zwischen ihrer Unterwerfung unter Natur oder der Natur unter das Selbst.“ (DdA 49) Was ist hier mit Unterwerfung gemeint? Ganz sicher nicht die simple Tatsache, dass sich Menschen zur Befriedigung elementarer Bedürfnisse absichtsvoll auf die äußere und innere Natur beziehen müssen. Damit die Geschichte der verhängnisvollen Zivilisation keine Brüche aufweist, sondern als Entfaltungsprozess eines identischen Prinzips erzählt werden kann, müssen selbst noch die frühen animistischen Kulturen als Ausdruck eines Herrschaftswillens interpretiert werden. Da Horkheimer und Adorno nicht Nietzsches ‚Wille zur Macht‘-Theorem übernehmen können, ohne den Versöhnungsgedanken aufzugeben, unterstellen sie, dass den frühen Menschen das Streben nach Naturbeherrschung (und darüber vermittelt das Streben nach sozialer Herrschaft) durch die Übermacht der Natur aufgezwungen wurde. Aber ist die Annahme haltbar? Jedenfalls widerspricht sie der ganzheitlichen Weltanschauung vieler archaischer Stämme. Das ‚wilde Denken‘ (Lévi-Strauss) kennt keine feindliche Natur, die zu unterwerfen wäre; vielmehr ordnet es alles, was ist, in einen unveränderlichen Gesamtzusammenhang ein. Dies zeigt sich bspw. im Totem, das die verwandtschaftliche Beziehung zwischen dem Einzelnen bzw. dem Clan und einer bestimmten Naturerscheinung darstellt, oder in archaischen Wirtschaftsformen, denen der Gedanke der Akkumulation völlig fremd ist (wie dies bei den Baruya der Fall war; siehe Godelier 1987, 184). Bei diesen Themen ist die empirische Forschung (Ethnologie, Anthropologie und Geschichtswissenschaft) ein besserer Ratgeber als die Philosophie. Die Naturauffassungen und Naturverhältnisse, die Identitätsvorstellungen und Triebhemmungen sowie die Formen sozialer Herrschaft und die spezifischen Bedingungen für ihr Entstehen (oder Ausbleiben) sind zu unterschiedlich, als dass sie sich in ein Schema pressen ließen, welches Animismus, Mythos, Polytheismus, Monotheismus, Metaphysik und Szientismus über den Leisten reprimierter Natur schlägt.

### Ein bemerkenswerter Briefwechsel

Horkheimers moralphilosophische Überlegungen sind vielschichtig. Auf der einen Seite reduziert er die Existenz von Moral soziologisch auf die Entzweiung in der bürgerlichen Gesellschaft, er weist die Ansprüche des Kognitivismus zurück – weder durch Intuition noch durch Argumentation könnten moralische Wahrheiten begründet werden –, präzisiert diese Kritik im Sinne des metaethischen Negativismus – wir wissen nicht, was ethisch gut ist, können aber angeben, was ethisch verwerflich ist (vgl. Horkheimer 1974, 215) –, unterfüttert diese Position durch die Idee des Mitleidens und versucht in ZK/DdA schließlich einen konstitutiven Zusammenhang von Vernunft und Herrschaft nachzuweisen. Auf der anderen Seite argumentiert er immer wieder auch freiheitstheoretisch, hedonistisch oder perfektionistisch; der Gedanke einer Vernunft, die sich selbst reflektiert und in der Folge ethische Ziele setzt, steht quer zum demonstrativen Nonkognitivismus. Auf dieser Linie liegt eine moralphilosophische Idee, die man bei Horkheimer eigentlich nicht erwarten würde: die Fundierung der Moral in Sprache. Am 4.9.1941, d.h. während der Arbeit an der *Dialektik der Aufklärung*, schreibt Horkheimer in einem Brief an Adorno:

Die Sprache intendiert, völlig unabhängig von der psychologischen Absicht des Sprechenden, auf jene Allgemeinheit, die man der Vernunft allein zugesprochen hat. Die Interpretation dieser Allgemeinheit führt notwendig auf die Idee der richtigen Gesellschaft. […] Die Rede an einen richten, heißt im Grunde, ihn als mögliches Mitglied des zukünftigen Vereins freier Menschen anerkennen. Rede setzt eine gemeinsame Beziehung zur Wahrheit, daher die innerste Bejahung der fremden Existenz die angeredet wird, ja eigentlich aller Existenzen ihren Möglichkeiten nach. Soweit die Rede die Möglichkeit verneint, befindet sie sich notwendig im Widerstreit mit sich selbst. (Horkheimer 1996, 171 f.)

Diese Idee einer kommunikationstheoretischen Grundlegung der Gesellschaftskritik, der Adorno in seinem nächsten Brief beipflichtet, wird Habermas ausarbeiten.

## Habermas: die sprachphilosophische Wende

Spätestens seit der *Dialektik der Aufklärung* sind bei Horkheimer und Adorno die Weichen so gestellt, dass die Frage nach den normativen Grundlagen der Gesellschaftskritik in einer (von ihnen offengehaltenen) Aporie mündet. Um dieses Begründungsdefizit zu beheben, nahm Habermas den Faden der Vernunftethik wieder auf. Sie sollte einerseits das universalistische, kognitivistische und prozedurale Erbe Kants antreten, andererseits nicht länger den ausgetretenen Pfaden der Bewusstseins‑/Subjektphilosophie folgen. Der Vernunftbegriff könne, so Habermas, nur sprechakttheoretisch, d.h. als Rekonstruktion der formalen Bedingungen möglicher Verständigung rehabilitiert werden (Formalpragmatik). Das in der ersten Generation der Kritischen Theorie nicht ausgewiesene Gegenstück zur instrumentellen Vernunft sei die kommunikative Vernunft: die in den lebensweltlich situierten natürlichen Sprachen fest verankerte Vernunft der Verständigung. „Verständigung wohnt als Telos der menschlichen Sprache inne.“ (TKH 387; nahezu identisch: SGS 211) Im Folgenden werde ich darlegen, dass Verständigung *nicht* der menschlichen Sprache als Telos innewohnt, sondern dass die Sprache Ausdruck der menschlichen Freiheit ist und insofern auch die *Möglichkeit* der Verständigung bereithält.

### Sprechen versus Handeln

Habermas zufolge gibt es zwei „elementare Handlungstypen, von denen sich der eine nicht auf den anderen reduzieren läßt“ (SGS 203). Die beiden Handlungstypen nennt er *Handeln im engeren Sinne* und *Sprechhandlungen*.

Handlungen im engeren Sinne, im exemplarischen Fall einfache nicht-sprachliche Tätigkeiten der erwähnten Art [Laufen, Aushändigen, Hämmern oder Sägen, S.E.], beschreibe ich als Zwecktätigkeiten, mit denen ein Aktor in die Welt eingreift, um durch die Wahl und den Einsatz geeigneter Mittel gesetzte Ziele zu realisieren. (SGS 197)

Sprachliche Äußerungen beschreibe ich als Akte, mit denen sich ein Sprecher mit einem anderen über etwas in der Welt verständigen möchte. (ebd.)

Bei Handlungen im engeren Sinne – Habermas verwendet auch die folgenden Synonyme: zweckrationale Handlungen, erfolgsorientierte Handlungen, teleologische Handlungen – werden *feststehende *„*egozentrische*“ (TKH 385) Ziele durch die Wahl effizienter Mittel verfolgt. Diese Handlungen können unter Beachtung technischer Regeln auf Gegenstände gerichtet sein (instrumentelles Handeln) oder sie können unter Beachtung der „Regeln rationaler Wahl“ der Beeinflussung eines „rationalen Gegenspielers“ (ebd.) dienen (strategisches Handeln). Die Rationalität des Handelns im engeren Sinne entspricht ziemlich genau der von Horkheimer beschriebenen instrumentellen Rationalität (der Unterschied ist terminologischer, nicht inhaltlicher Natur; Habermas binnendifferenziert lediglich die instrumentelle Vernunft Horkheimers in gegenständliches instrumentelles Handeln und soziales strategisches Handeln).

Anders verhalte es sich bei den Sprechhandlungen. Mit ihnen wird kein feststehender egoistischer Zweck verfolgt, sondern *Verständigung* angestrebt. Das bedeutet nicht, dass Sprechhandlungen nach Habermas zweckfrei wären – wobei dieses Missverständnis sehr naheliegend ist, da er Verständigungsrationalität von Zweckrationalität scharf abgrenzt. Das wesentliche am verständigungsorientierten Sprechen ist vielmehr, dass die Ziele *vorbehaltlos* und *einvernehmlich* festgelegt werden: Die Beteiligten sind „nicht primär am eigenen Erfolg orientiert“ (ebd.), sondern erzielen durch das wechselseitige Erheben und Prüfen von Geltungsansprüchen einen begründeten Konsens. Bei der Koordinierung ihrer Willen unterwerfen sie sich also dem zwanglosen Zwang des besseren Arguments, d.h. sie passen nicht das Argument der individuellen Interessenlage an, sondern lassen sich von interpersonell als gültig anerkannten Gründen leiten. Damit ist zugleich klar, dass Sprechhandlungen nicht nur in Kooperationszusammenhänge eingelassen sein *können*, sondern immer schon eine dreifache Relation voraussetzen: *Sprecher* und *Hörer* verständigen sich *über etwas in der Welt*.

Soweit die terminologische Festlegung. Aber ist sie auch plausibel? Kontraintuitiv erscheint zunächst, dass Handlungen – im Unterschied zu Sprechhandlungen – egozentrisch motiviert sein sollen. Wer ein Bild aufhängt (instrumentelles Handeln), kann dies für sich tun, er kann aber auch seinem Lebensgefährten eine Freude machen wollen. Das gleiche gilt für das strategische Handeln. Dies kann, *muss* aber nicht egozentrisch sein. Es gibt Situationen, in denen es durchaus geboten ist, altruistische Ziele strategisch zu verfolgen. Selbstverständlich lässt sich zwischen monologisch gefassten feststehenden Zielen und vorbehaltlos-konsensual festgelegten Zielen unterscheiden, aber die Verknüpfung feststehender Ziele mit der „zweckrationalen Ausrichtung am jeweils eigenen Erfolg“ (SGS 218) überzeugt nicht. Lässt man diese Verknüpfung jedoch fallen, stellt sich die Frage, ob die Sprechhandlung, wie Habermas behauptet, wirklich ein irreduzibler, d.h. nicht in Zweckrationalität aufgehender Handlungstyp ist. Habermas bemüht drei Argumente für seine Behauptung:

#### Argument 1:

Die Sprechhandlung sei ein eigenständiger Handlungstyp, weil ihr ein eigenständiger *Rationalitätstyp* zugrunde liege: die kommunikative Rationalität. Warum? Sprechakte haben nach Habermas anderen Rationalitäts*bedingungen* zu genügen als gewöhnliche Handlungen (vgl. SGS 202). Sprechakte seien genau dann rational, wenn sie *wahr, richtig *und* wahrhaftig* sind, während zweckorientierte Handlungen rational seien, wenn *das Ziel effizient erreicht wird*.

#### Argument 2:

Sprechhandlungen kommentieren sich implizit selbst und geben dergestalt die Intention des Sprechers zu erkennen. Bei den Handlungen im engeren Sinne sei das nicht der Fall. Ich könne aus der Beobachterperspektive zwar die Handlung einer anderen Person identifizieren – sie läuft die Straße entlang –, aber die der Handlung zugrundeliegende Intention lässt sich der Handlung selbst nicht entnehmen (die Person kann verschiedene Gründe haben, die Straße entlang zu gehen). Im Vergleich dazu seien Sprechhandlungen „nicht in demselben Sinne interpretationsbedürftig“ (SGS 199). Wenn beispielweise Person X der Person Y den Befehl gibt, die Waffe fallen zu lassen, dann wisse der Hörer des Sprechaktes – anders als der Beobachter des Fußgängers – genau, um welche Handlung es sich gerade handelt: um einen Befehl.

#### Argument 3:

Sprechhandlungen unterscheiden sich von gewöhnlichen Handlungen „durch die Art der Erfolge, die durchs Sprechen erreicht werden können“ (SGS 199). Das Handlungsziel der Zwecktätigkeiten sei (a) unabhängig von den eingesetzten Mitteln, (b) ein kausal zu bewirkender Zustand und (c) ein Zustand in der objektiven Welt. Anders verhalte es sich bei Sprechhandlungen.

(a) Sprechhandlungen haben zwei illokutionäre^10^ Ziele: Sie sollen zum einen verstanden, zum anderen als gültig anerkannt werden (als wahr, richtig, wahrhaftig). Diese Ziele können „nicht unabhängig von den linguistischen Mitteln der Verständigung definiert werden“ (SGS 200).

(b) Der Sprecher kann seine Ziele nicht erzwingen, „weil der illokutionäre Erfolg von der rational motivierten Zustimmung des Hörers abhängt – Einverständnis in der Sache muß ein Hörer durch die Anerkennung eines kritisierbaren Geltungsanspruchs gleichsam aus freien Stücken besiegeln. Illokutionäre Ziele sind nur kooperativ zu erreichen“ (SGS 201).

(c) Die Perspektive der Zwecktätigkeit kennt nichts anderes als manipulierbare Objekte, aber im Sprechakt begegnen sich Sprecher und Hörer „als Angehörige der intersubjektiv geteilten Lebenswelt“ (SGS 201).

#### Anm. zu Argument 1:

Gegen die Behauptung, dass sich Sprechhandlungen und Handlungen im engeren Sinne dahingehend unterscheiden, dass sie unterschiedlichen Rationalitätsbedingungen genügen müssen, sprechen folgende Überschneidungen: Zum einen lässt sich nicht nur das erfolgsorientierte, sondern auch das verständigungsorientierte Handeln unter dem Gesichtspunkt der Effizienz thematisieren – schlicht weil Verständigung selbst ein Ziel ist und als Mittel der Handlungskoordination eingesetzt wird (vgl. Steinhoff 2015, 72) –, zum anderen liegen erfolgsorientierten Handlungen Annahmen über Objekte und deren *causal powers* zugrunde, die sich als wahr oder unwahr erweisen können. Im Falle einer technischen Handlung einer Einzelperson kann das propositionale Wissen nur nicht die Gestalt eines diskursiv begründbaren Geltungsanspruches annehmen, weil keine weiteren Personen im Spiel sind, an die sich ein Behauptungsakt richten ließe – nichtsdestotrotz ist der Erfolg der Handlung abhängig von der *Begründetheit* der Annahmen (sehen wir vom Fehlschluss der Scheinkausalität mal ab). Angesichts dieser Überschneidungen zwischen erfolgsorientierter und verständigungsorientierter Handlung liegt die Vermutung nahe, dass diese ein *Anwendungsfall* oder eine *Spezifikation* jener ist – aber eben keinen eigenständigen Handlungstyp bildet.^11^ Diese Vermutung erhärtet sich, wenn man die Behauptung von Habermas hinterfragt, Sprechhandlungen seien nur dann rational, wenn sie wahr, richtig und wahrhaftig sind. Gibt es nicht viele Sprechakte, in denen der Sprecher unwahrhaftig ist und trotzdem rational handelt? Ich denke hier keineswegs nur an egozentrisch motivierte Lügen. Häufig sind Sprecher bewusst unwahrhaftig, haben aber das Wohl des Hörers oder das Wohl anderer im Sinn. Allgemein gesagt: Die Rationalität eines Sprechaktes ist wie die Rationalität *aller* Handlungen abhängig von der Beschaffenheit des anvisierten Ziels. Ist das Einverständnis in der Sache Bestandteil des anvisierten Ziels, müssen die Präsuppositionen der argumentativen Rede eingehalten werden – eine Lüge wäre *in diesem Fall* (aber eben auch *nur* in diesem Fall) irrational. Um die These zu verteidigen, dass „[z]weckorientierte Interventionen und Sprechakte […] jeweils anderen Bedingungen der Rationalität“ (SHS 202) genügen, müsste Habermas nachweisen können, dass nicht nur eine besondere Gattung von Sprechakten, sondern der Sprechakt *als solcher* ein verständigungsorientiertes Ziel hat (weshalb alle Sprechakte, die dieses Ziel nicht verfolgen, pervertierte Sprechakte sind).

#### Anm. zu Argument 2:

Bereits die von Habermas verwendete Formulierung, Sprechakte seien nicht „in demselben Sinne interpretationsbedürftig“, weist darauf hin, dass zwischen sprachlichen und nicht-sprachlichen Handlungen kein grundlegender, sondern höchstens ein *gradueller* Unterschied besteht, denn Sprechakte müssen vom Hörer ebenfalls interpretiert werden. So muss der Hörer auf die Tonlage des Sprechers achten, seine Gestik und Mimik berücksichtigen und den situativen Kontext einbeziehen, um das Gesagte nicht falsch zu verstehen – kurz, er muss *beobachten* und auf Grundlage der so gewonnen *Eindrücke* auf die Intention *schließen*.

#### Anm. zu Argument 3:

(a) Dass sich bei der Sprechhandlung das intendierte Ziel (Verständigung) nicht unabhängig vom Mittel (Grammatik) erreichen lässt, *ist kein Spezifikum des Sprechakts*. Das gleiche gilt für viele erfolgsorientierte Handlungsziele, sobald diese nur hinreichend konkret gefasst werden. Bio-Joghurts z.B. (Ziel) lassen sich nur durch den Einsatz von Bio-Zutaten (Mittel) gewinnen.

(b) Es ist richtig, dass sich der *illokutionäre* Erfolg von Sprechakten (Verstehen des Gesagten und Anerkennung der erhobenen Geltungsansprüche) nicht kausal bewirken lässt. Ich kann nicht erzwingen, dass man mich versteht und meine Behauptung für wahr, richtig und wahrhaftig hält. Jedoch – und das ist Habermas durchaus klar – hängt der *perlokutionäre* Erfolg des Sprechens nicht zwingend davon ab, dass *alle* Geltungsansprüche, die erhoben werden können, auch tatsächlich erhoben und vom Hörer aus freien Stücken als gültig akzeptiert werden; der perlokutionäre Erfolg kann – ein entsprechendes Drohpotential vorausgesetzt – genauso gut erzwungen sein.

(c) Wenn es Sprechakte gibt, in denen dem Hörer kaum eine andere Wahl gelassen wird, als das zu tun, was der Sprecher verlangt, dann gibt es eben doch Sprechakte, in denen der Hörer zum Objekt der Beeinflussung degradiert wird. Nun ist es nicht so, dass Habermas dies nicht wüsste. Allerdings geht er anscheinend davon aus, zwischen *eigentlichem *und* uneigentlichem* Sprechen begründet unterscheiden zu können und deshalb die grundlegende Differenz von Sprechhandlungen und Handlungen im engeren Sinne nicht aufgeben zu müssen:

Nun bietet ersichtlich nicht jede sprachlich vermittelte Interaktion ein Beispiel für verständigungsorientiertes Handeln. Die elementare Sprechhandlung kann nur dann als Modell für eine nicht ihrerseits auf erfolgsorientiertes Handeln zurückführbare Konsenshandlung dienen, wenn sich der auf Verständigung gerichtete Sprachgebrauch als der Originalmodus von Sprachverwendung überhaupt auszeichnen läßt, zu dem sich der konsequenzenorientierte Sprachgebrauch und die indirekte Verständigung (das Zu-verstehen-Geben) parasitär verhalten. (Habermas 1984, 595 f.)

Den Nachweis, dass das strategische Sprechen vom kommunikativen Sprechen abhängig und deshalb die normativ gehaltvolle Verständigungsorientierung der Originalmodus von Sprache sei, möchte Habermas in seiner Interaktionstheorie antreten.

### Kommunikatives versus strategisches Handeln

Interaktionen dienen der Handlungskoordination. Handlungen müssen koordiniert werden, sobald sich der Handlungsplan eines Aktors nur durch die Hilfe oder die Unterlassung eines anderen Aktors umsetzen lässt. Bei Interaktionen gehen die beiden Handlungstypen (verständigungsorientiertes und erfolgsorientiertes Handeln) eine Verbindung ein – und je nachdem, welcher Handlungstyp in dieser Verknüpfung dominiert, gestaltet sich der Charakter der Interaktion. Das soziale Handeln ist entweder *kommunikativ* oder es ist *strategisch*, „je nachdem, ob die illokutionären Kräfte von Sprechakten eine handlungskoordinierende Rolle übernehmen oder ob die Sprechhandlungen ihrerseits der außersprachlichen Dynamik von Einflußnahmen zwecktätig aufeinander einwirkender Aktoren derart subordiniert werden, daß die spezifisch sprachlichen Bindungsenergien ungenutzt bleiben.“ (SGS 203) Während beim kommunikativen Handeln Sprache *als Sprache*, d.h. als „Quelle der sozialen Integration“ (SGS 204) in Anspruch genommen wird, stutzt das strategische Handeln Sprache auf die Übermittlung von Informationen zurecht und verfehlt damit ihr normatives Telos.

Habermas verdeutlicht dies am *latent strategischen Handeln*. Sein Beispiel (vgl. SGS 206): Sprecher S fordert den Hörer H auf, Y Geld zu geben. S verschweigt H jedoch, dass Y das Geld benötigt, um eine Straftat vorzubereiten, von der S annimmt, dass H sie nicht billigen und deshalb Y kein Geld geben würde. Mit anderen Worten: H handelt nur dann im Sinne von S, wenn dieser vorgibt, dass er seine illokutionären Ziele ohne „egozentrisch[e]“ (SGS 207) Hintergedanken verfolgt (was aber nicht der Fall ist). Der latent-strategische Sprechakt gaukelt also vor, etwas anderes zu sein, als er ist, nämlich ein gewöhnlicher verständigungsorientierter Sprechakt. Daran zeigt sich nach Habermas, was der verständigungsorientierte Sprachgebrauch präsupponiert: die subjektive Wahrhaftigkeit des Sprechenden. Das latent strategische Sprechen „lebt parasitär vom normalen Sprachgebrauch, weil er nur dann funktioniert, wenn mindestens eine Seite davon ausgeht, daß die Sprache verständigungsorientiert gebraucht wird.“ (SGS 207) Die Lüge und das Verschweigen relevanter Informationen sind lediglich „abgeleitete“ (SGS 207) Sprechakte; sie sind *Derivate* von normalen Sprechhandlungen, in denen der Sprecher vom propositionalen Gehalt und der normativen Richtigkeit subjektiv überzeugt ist.

Vom *latent* strategischen Gebrauch der Sprache unterscheidet Habermas den *manifest* strategischen Sprechakt, der sich dadurch auszeichnet, dass der Anspruch normativer Gültigkeit durch einen sanktionsbewehrten Imperativ ersetzt wird. Habermas veranschaulicht dies am „Hände hoch!“ des Bankräubers. Bei der unverhohlenen Drohung mit der Waffe handele es sich um einen „illokutionär entkräfteten“ Sprechakt; die Sprache sei „depotenziert“, weil sie lediglich der Übermittlung von Informationen, nicht jedoch der „Konsensbildung“ (SGS 209) diene. „Solche perlokutionär verselbständigten Akte sind überhaupt keine illokutionären Akte, denn sie zielen nicht auf die rational motivierte Stellungnahme eines Adressaten.“ (SGS 209 f.)

#### Anm. zum latent strategischen Sprechakt

Das Parasitismus-Argument ist keineswegs neu; der Sache nach findet es sich schon bei Kant. Dieser hatte darauf hingewiesen, dass die lügenhafte Maxime, verallgemeinert gedacht, sich selbst zerstöre, weil sie die Bedingung ihrer Möglichkeit untergrabe: Wenn sich jeder die Lüge vorbehalten würde, schenkte niemand mehr dem anderen glauben, womit die (nun erwartete) Lüge jeden Sinn verlöre. Wahrhaftig zu sein, sei eine „Regel, die ihrem Wesen nach keiner Ausnahme fähig ist, weil sie sich in dieser geradezu selbst widerspricht.“ (Kant 1907 [1797], 430) Die lügenhafte Maxime – bei Habermas: das verdeckt strategische Sprechen – sei somit unselbständig. Es hat den Anschein, dass Habermas seinem Ziel, Verständigung als Originalmodus der Sprache nachzuweisen, damit ein ganzes Stück nähergekommen ist. Um es zu erreichen, müsste er allerdings noch zeigen, dass auch das offen strategische Handeln ein Derivat des kommunikativen Handelns ist.

#### Anm. zum manifest strategischen Sprechakt

Habermas schwankt zwischen der Aussage, beim offen strategischen Handeln würden nicht alle Möglichkeiten der Sprache genutzt – die Sprache sei „depotenziert“ – und der weitergehenden Aussage, machtgestützte Imperative seien „überhaupt keine illokutionären Akte“ (SGS 209). Zutreffend ist nur ersteres, letzteres nicht (vgl. Köveker 1992, 303). Dies wird deutlich, wenn wir die Implikationen des Sprechakts des Bankräubers sprachlich explizit machen. Er sagt nun zum Schalterbeamten: „Hände hoch! Die hier [zeigt auf die Pistole] ist kein Spielzeug. Sollte ich nicht kriegen, was ich will, werde ich von ihr Gebrauch machen. Ich weiß übrigens, dass der Tresor randvoll ist, also bescheiß’ mich nicht, wenn Dir Dein Leben lieb ist.“ Nehmen wir an, der Bankangestellte versteht die Äußerung. Nehmen wir ferner an, er glaubt dem Bankräuber, dass die Waffe keine Attrappe ist und er sie auch einsetzen würde. Weil ihm sein Leben lieb ist, händigt er das Geld aus. Diesen perlokutionären Erfolg erzielt der Bankräuber nur, weil er gleich mehrere illokutionäre Erfolge erzielt hat: Seine Sätze sind verstanden worden, die Wahrheit der assertorischen Sätze „Diese Waffe ist kein Spielzeug“ und „Ich weiß, dass der Tresor randvoll ist“ wurde als gültig anerkannt und auch die Wahrhaftigkeit der Ankündigung, die Pistole im Zweifelsfall zu benutzen, ist nicht infrage gestellt worden. Der einzige illokutionäre Erfolg, den der Bankräuber nicht erzielen konnte – aber eben auch nicht erzielen *wollte* –, betrifft die normative Richtigkeit.

Weil der Bankräuber zwei der drei möglichen Geltungsansprüche erheben muss, um an die Beute zu kommen, erweist sich auch die Behauptung als unzutreffend, dass die Androhung von Gewalt „nicht auf die rational motivierte Stellungnahme eines Adressaten“ zielt. Der Bankangestellte prüft im Rahmen seiner Möglichkeiten Proposition und Wahrhaftigkeit. Er wägt dann *rational* zwischen den Folgen zweier *Stellungnahmen* ab: der Weigerung und dem Folgeleisten.

Für die These von Habermas, Sprache an sich habe ein normatives Telos, ist der folgende Punkt entscheidend: Während der verdeckt strategische Sprachgebrauch nur unter der Bedingung erfolgreich sein kann, dass der Hörer dem Sprecher irrtümlich unterstellt, verständigungsorientiert zu sein, lässt sich das offen strategische Handeln *nicht* auf kommunikatives Handeln zurückführen. Das Parasitismus-Argument versagt an dieser Stelle, weil das offen strategische Sprechen überhaupt nicht vorgibt, etwas anderes zu sein, als es ist. Weil es keinen Anspruch auf normative Richtigkeit erhebt, ist es lediglich genügsamer – diese Genügsamkeit macht es aber nicht zu einem Derivat des kommunikativen Handelns. In der TKH hat Habermas dies zwischenzeitlich selbst so gesehen: Die offen strategische, sich auf Sanktionen oder Gratifikationen stützende Aufforderung wird als eine eigene Sprechaktklasse von den „*normativ autorisierten* Aufforderungen“ (vgl. TKH 404) unterschieden. Die Anweisung der Stewardess, sich beim Landeanflug anzuschnallen, ist normativ autorisiert, weil sie auf allgemein akzeptierten und gesetzlich verankerten Sicherheitsbestimmungen beruht; die nachdrückliche Aufforderung des Arztes, mit dem Rauchen umgehend aufzuhören, ist insofern normativ autorisiert, als er eine fachliche Ausbildung absolviert hat, über eine staatliche Genehmigung verfügt (Approbation) und der Zusammenhang des Rauchens mit schweren Erkrankungen allgemein anerkannt ist. Stewardess und Arzt können sich auf *allgemein rechtfertigbare Gründe* berufen. Dieser Abgrenzung der normativ autorisierten Imperative von den einfachen oder machtbasierten Imperativen stehen jedoch schon in der TKH Passagen entgegen, die nahelegen, dass nicht nur das verdeckte, sondern auch das offen strategische Sprechen parasitär sei (siehe Köveker 1992, 291 f.). In der TKH schwankt Habermas also zwischen der Position, das offen strategische Sprechen sei eine selbständige und damit nicht-parasitäre Sprechaktklasse, und der damit inkompatiblen Annahme, eine wesentliche Gemeinsamkeit von offen und verdeckt strategischen Sprechhandlungen sei ihr Parasitismus. Im Zuge seiner Auseinandersetzung mit Kritiken – insbesondere denen von Erling Skjei (1985) und Ernst Tugendhat (1985) – wird sich Habermas für die zweite Position entscheiden und damit seine scharfe Abgrenzung machtbasierter und normativ berechtigter Aufforderungen revidieren. Fortan ordnet er offen strategische Handlungen den normativ autorisierten Handlungen als parasitären Extremfall zu:

Es war nun mein Fehler, diesen Grenzfall des reinen, machtgestützten Imperativs als eine eigene Klasse von Sprechakten zu behandeln. […] Ich habe in meiner Antwort auf Skjei bereits eine Revision vorgenommen: ich betrachte heute einfache oder normativ nichtautorisierte Aufforderungen als parasitären Fall. Als Soziologe hätte ich wissen müssen, daß ein Kontinuum zwischen der bloß faktisch eingewöhnten und der in normative Autorität verwandelten Macht besteht. Deshalb lassen sich alle Imperative, denen wir eine illokutionäre Kraft zuschreiben, nach dem Muster normativ autorisierter Aufforderungen analysieren. Was ich fälschlicherweise für einen kategorialen Unterschied gehalten habe, schrumpft zu einem graduellen. Die durchs „Hände hoch!“ sanktionierte Aufforderung des Bankräubers gehört zu jenen Grenzfällen eines manifest strategischen Gebrauchs von Sprechhandlungen, bei dem die fehlende illokutionäre Kraft durch Berufung auf ein Sanktionspotential ersetzt wird. Parasitär ist dieser Gebrauch insofern, als das Verständnis eines solchen Sprechakts den Verwendungsbedingungen für normativ autorisierte, nicht-depravierte Aufforderungen entliehen wird. (Habermas 1986, 361 f.)

Bereits die Zuordnung der offen strategischen Sprechakte zur Kategorie der normativ autorisierten Sprechakte ist alles andere als überzeugend. Um eine Analogie aus dem Bereich der Handlungen im engeren Sinne zu bemühen: Wenn der offen strategische Sprechakt, der gar keinen Anspruch auf normative Richtigkeit erhebt, ein Grenzfall der normative Richtigkeit reklamierenden Sprechakte sein soll, dann ist eine Person, die ihr Leben lang kein Interesse an Eis gezeigt hat, auch ein Grenzfall des Eisliebhabers. Wie verhält es sich aber mit der Aussage, offen strategische Sprechakte seien insofern parasitär, als der Hörer die machtgestützte Aufforderung des Sprechers nur deshalb *verstehen* könne, weil er ein implizites Wissen normativ autorisierter Sprechakte mitbringe? Gehen wir die einzelnen Präsuppositionen der Rede durch. Wie gezeigt, weisen die offen strategische und die kommunikative Sprechhandlung in propositional-konstativer und expressiv-intentionaler Hinsicht Überschneidungen auf: Weil der offen strategische Sprechakt den Anspruch auf Wahrheit und Wahrhaftigkeit erhebt, muss dessen Adressat auch keine Anleihen bei kommunikativen Sprechhandlungen machen, um ihn zu verstehen. „Wir verstehen eine Sprechhandlung, wenn wir die Art von Gründen kennen, die ein Sprecher anführen könnte, um einen Hörer davon zu überzeugen, daß er unter den gegebenen Bedingungen berechtigt ist, Gültigkeit für seine Äußerung zu beanspruchen – kurz, wenn wir wissen, was sie akzeptabel macht.“ (SGS 210) Angewendet auf unser Beispiel: Der Bankangestellte versteht den Bankräuber in konstativer und expressiver Hinsicht, weil er weiß, was dieser tun müsste, um ihn davon zu überzeugen, dass er berechtigt ist, Gültigkeit für seine Äußerung zu beanspruchen. Der Bankräuber könnte beispielsweise eine Geisel erschießen, um die Echtheit seiner Waffe und die Aufrichtigkeit seiner Ankündigung unter Beweis zu stellen.^12^ Und wie ist es um die Präsupposition der normativen Richtigkeit bestellt? Habermas schlägt offenbar vor, normativ *ungedeckte* Sprechhandlungen (wie „Hände hoch!“) dem Kontinuum normativ *gedeckter* Sprechhandlungen (wie „Zahlen sie mir 100 Euro von meinem Konto aus“) zuzuschlagen, um dann behaupten zu können, dass jene nur deshalb verstanden werden können, weil der Hörer mit den normativ gedeckten Sprechhandlungen – zu denen die offen strategischen Sprechhandlungen als Grenzfall nun auch gehören sollen – bereits vertraut ist. Aber wie gesagt, die Drohung und das unmoralische Angebot sind keine Sprechakte, die vorgeben, normativ richtig zu sein. Deshalb muss für ihr Verständnis den normativ gedeckten Sprechhandlungen auch nichts entliehen werden. Mit anderen Worten: Sie sind nicht parasitär. Um die These zu retten, dass die kommunikative Sprechhandlung der Originalmodus von Sprache ist, müssten sie es aber sein.

Indem Habermas behauptet, kommunikative Sprechakte seien der Originalmodus von Sprache, konfundiert er letztlich Gattung und Art. Genauso, wie sich die Gattung der Lebewesen in die Arten der vernunftbegabten Lebewesen (Menschen) und der nicht-vernunftbegabten Lebewesen (Tiere) differenzieren lässt, kann der Sprachgebrauch (Gattung) in verständigungsorientierten und strategischen Sprachgebrauch (Arten) unterschieden werden. Übertragen wir die Behauptung von Habermas, dass selbst noch das offen strategische Sprechen ein Derivat verständigungsorientierten Sprechens sei, auf die Gattung der Lebewesen, liefe dies auf die These hinaus, dass Tiere uneigentliche Menschen sind und die menschliche Vernunft dem Leben als Telos innewohnt. Dies ist aber nicht der Fall. Und ebenso hat auch Sprache kein normatives Telos. Ähnlich wie sittliche Institutionen bietet sie als sedimentierter Ausdruck menschlicher Freiheit jedoch die *Möglichkeit* der vorbehaltlosen und darum sozialintegrativen Handlungskoordination.

### Diskursethik

Die starke und darum interessante These, Sprache an sich habe ein normatives Telos, mag nicht überzeugen, jedoch stellt sich die Frage, welche Bedeutung ihr für die Ausarbeitung der Diskursethik zukommt. Um es kurz zu machen: gar keine. In seinem klassischen und nach wie vor maßgeblichen Aufsatz *Diskursethik. Notizen zu einem Begründungsprogramm* beansprucht Habermas nämlich nicht, sein Moralprinzip – den Universalisierungsgrundsatz (U) – aus den unausweichlichen Voraussetzungen der Rede schlechthin abzuleiten, sondern aus den Präsuppositionen der *argumentativen* Rede zu entwickeln. Der von K.-O. Apel und W. Kuhlmann übernommene Grundgedanke lautet, dass jeder, der ernsthaft argumentiert, implizit bereits Diskursregeln anerkannt hat, deren explizite Verneinung zu einem performativen Widerspruch führe. In Kurzform: Jeder Sprecher, der argumentiert, bezieht sich auf unausweichliche Diskursregeln (implizites Wissen), aus denen sich, sobald sie mittels der Methode des performativen Widerspruchs zu explizitem Wissen transformiert wurden, das gesuchte Prinzip der Moral ergibt (Argumentation > Diskursregeln > Moralprinzip).

Im Folgenden werde ich die Diskursethik von Habermas nicht eingehend behandeln können. Sei es die Sinnhaftigkeit/Reichweite des Arguments vom performativen Widerspruch, der genaue Inhalt der Diskursregeln, die unterschiedlichen Interpretationsmöglichkeiten des Universalisierungsgrundsatzes, das Verhältnis von Universalisierungsgrundsatz und Diskursprinzip, das Verhältnis von Moral und Recht, die Überführung der Prinzipien in empirische Diskurse etc. – die Diskursethik von Habermas sieht sich vielen Fragen ausgesetzt, die den Rahmen meines Beitrags sprengen würden. Ich werde mich deshalb auf ein begründungstheoretisches Kernproblem konzentrieren: Selbst wenn wir für einen kurzen Moment annehmen, dass sich für die unausweichlichen Argumentationspräsuppositionen, die Habermas im Anschluss an R. Alexy angibt, schlüssig argumentieren ließe, lässt sich dann auch nachweisen, dass der Universalisierungsgrundsatz aus ihnen folgt? Lässt sich zeigen, „*wie das […] Verallgemeinerungsprinzip von Voraussetzungen der Argumentation überhaupt impliziert wird*“? (Habermas 2009, 86)

Dafür müssen wir uns die Prämissen und das Ziel der Begründung genauer ansehen. Ziel ist die Deduktion des Universalisierungsgrundsatzes, der, ähnlich wie Kants kategorischer Imperativ, ein Prüfverfahren für konkrete Normen darstellt:

So muß jede gültige Norm der Bedingung genügen, daß die Folgen und Nebenwirkungen, die sich jeweils aus ihrer *allgemeinen* Befolgung für die Befriedigung der Interessen eines *jeden* Einzelnen (voraussichtlich) ergeben, von *allen* Betroffenen akzeptiert (und den Auswirkungen der bekannten alternativen Regelungsmöglichkeiten vorgezogen) werden können. (Habermas 2009, 60)

Zu den Prämissen der Deduktion gehören die Diskursregeln. Habermas geht zwar auf verschiedene Diskursregeln ein, verwendet für die Ableitung des Universalisierungsgrundsatzes aber lediglich die folgenden:

(3.1.) Jedes sprach- und handlungsfähige Subjekt darf an Diskursen teilnehmen.
(3.2.) a. Jeder darf jede Behauptung problematisieren.
  b. Jeder darf jede Behauptung in den Diskurs einführen.
  c. Jeder darf seine Einstellungen, Wünsche und Bedürfnisse äußern.
(3.3.) Kein Sprecher darf durch innerhalb oder außerhalb des Diskurses herrschenden Zwang daran gehindert werden, seine in (3.1.) und (3.2.) festgelegten Rechte wahrzunehmen. (ebd., 89 f.)

Die entscheidende Frage lautet: Liegt hier tatsächlich eine materiale Implikation vor? Folgt das Moralprinzip U logisch aus den Diskursregeln? Offensichtlich nicht. Aus den egalitären Voraussetzungen der argumentativen Rede folgt kein wie auch immer geartetes Moralprinzip. Tugendhat (1993, 168) veranschaulicht dies an einem Potentaten, der seinen Untergebenen alle Rederechte zubilligt (3.1.-3.3.), die Normen aber festlegt, wie es ihm beliebt. Die Feststellung, er sei dazu gar nicht berechtigt, weil nur solche Normen gültig seien, die von Gleichgestellten akzeptiert werden, würde bereits die Geltung von U voraussetzen, womit die Argumentation in einem Zirkel mündete.

Habermas hat dies selbst so gesehen. Obgleich seine Ankündigung anderes vermuten ließ, leitet er das Verallgemeinerungsprinzip nicht im Sinne Apels rein transzendentalpragmatisch aus den Diskursregeln ab, sondern er fügt noch eine zweite Prämisse ein. Die Deduktion erfolge „in Verbindung mit der Idee der Rechtfertigung von Normen“ (ebd., 99; ähnlich: 94); erst wenn man angebe, „was es heißt, eine Handlungsnorm zu rechtfertigen“ (ebd., 87), könne man in Kombination mit den Diskursregeln zu U gelangen. Was verbirgt sich hinter der Idee der Rechtfertigung? In der ersten, in *Moralbewußtsein und kommunikatives Handeln* veröffentlichten Auflage erläutert Habermas sie wie folgt:

Wenn jeder, der in Argumentationen eintritt, u.a. Voraussetzungen machen muß, deren Gehalt sich in Form der Diskursregeln (3.1.) bis (3.3.) darstellen läßt; *und wenn wir ferner mit gerechtfertigten Normen den Sinn verbinden, daß diese gesellschaftliche Materien im gemeinsamen Interesse der möglicherweise Betroffenen regeln*, dann läßt sich jeder, der den ernsthaften Versuch unternimmt, normative Geltungsansprüche diskursiv einzulösen, auf Verfahrensbedingungen ein, die einer impliziten Anerkennung von U gleichkommen. (Habermas 1983, 103; Herv. von mir)

Bei dem von mir hervorgehobenen Satzteil, der die Idee der Rechtfertigung präzisiert, soll es sich um eine Prämisse handeln, die zusammen mit den Diskursregeln der Deduktion von U dient. Es ist jedoch offensichtlich, dass der Satzteil lediglich eine *Paraphrase* von U darstellt; die zweite Prämisse ist also bedeutungsgleich mit der Konklusion. Damit liegt keine Ableitung vor, sondern eine Tautologie, bei der die Diskursregeln übrigens keine Rolle mehr spielen. In Kurzform sagt Habermas: Aus den Diskursregeln und U folgt U (vgl. Tugendhat 1993, 169; Lumer 1997). Dass sein Argument petitiös ist, hat Habermas rückblickend eingestanden:

Allerdings darf die Idee der Rechtfertigung von Normen nicht zu stark sein und nicht schon das in die Prämisse einführen, worauf doch erst geschlossen werden soll: daß gerechtfertigte Normen die Zustimmung aller Betroffenen finden können. Dieser Fehler ist mir unterlaufen in J. Habermas (1983, 102 f.); er wurde in der zweiten Auflage (1985) korrigiert. (Habermas 1991a, 13, Fn. 7)

Die Korrektur besteht darin, die den Universalisierungsgrundsatz bereits enthaltende Idee der Rechtfertigung durch einen „schwachen, d.h. nicht-präjudizierenden Begriff von Normenrechtfertigung“ (Habermas 2009, 94) zu ersetzen. Der oben zitierte und von mir hervorgehobene Satzteil lautet nun: „und wenn wir ferner wissen, was es heißt, hypothetisch zu erörtern, ob Handlungsnormen in Kraft gesetzt werden sollen; dann […]“ (ebd.). Was sich hinter dem abgeschwächten Verständnis der Normrechtfertigung genau verbirgt, wird an Ort und Stelle leider nicht erörtert. In dem Aufsatz *Erläuterungen zur Diskursethik* findet sich allerdings die Bemerkung, „der (schwache) Sinn“ sei, dass „moralische Begründungen einen Dissens über Rechte und Pflichten […] auflösen“ (Habermas 1991b, 134). Die Begründung des Moralprinzips, so auch C. Lumer (1997), ist damit zwar nicht mehr petitös, aber sie versetzt uns gewissermaßen zum Anfangsproblem zurück. Denn die zweite Prämisse ist nun derart schwach, dass sie auch in Kombination mit den Diskursregeln nicht zu U führt. Dieses für seine Diskursethik entscheidende Begründungsproblem, das ihn erst zur Aufnahme der starken Idee der Rechtfertigung in der ersten Auflage veranlasste – in einem Manuskript des Aufsatzes aus dem Jahr 1982 wurde U noch rein aus den Diskursregeln abgeleitet (vgl. Lumer 1997) –, hat Habermas bis heute nicht gelöst.

## Kritische Theorie heute

Die geschichtsphilosophischen Kernaussagen von Horkheimer und Adorno werden nur noch selten offensiv verteidigt.^13^ Martin Saar, der profilierteste Frankfurter Fürsprecher genealogischer Kritik, ist eher an mehrdimensionalen Analysen sozialer Machtverhältnisse im Anschluss an die Studien Foucaults interessiert denn an der Wiederaufnahme des eindimensionalen, am Begriff des Selbsterhalts aufgezogenen Dialektik-Projekts. Bereits in seiner Dissertation monierte er an Nietzsche die „maßlose und pauschale Kritik der Moral der Gegenwart“ (Saar 2007, 18 f.). Nietzsche interessiert ihn erklärtermaßen weniger aus sachlichen denn aus *methodischen* Gründen. Sein Fokus liegt auf dem „wirkungsvollen Verfahren“ (ebd.) der Befragung von Selbstverständlichkeiten; an Nietzsche könne man v.a. lernen, wie sich das, was bislang fraglos galt, auf Distanz bringen lasse.^14^ Andernorts geht Saar zwar detailliert auf die inhaltlichen und rhetorischen Entsprechungen zwischen Nietzsches *Genealogie der Moral* und der *Dialektik der Aufklärung* ein, hält sich mit einer Bewertung ihrer Hauptaussagen aber weitgehend zurück. Die Bemerkung, letztere „häufe Ambivalenzen und Mehrdeutigkeiten aufeinander und weist keinen Ausweg“ (Saar 2017, 161), belegt indes gewisse Vorbehalte.

Auch wenn die Kritik, die Habermas am einseitigen Moderne-Verständnis von Horkheimer und Adorno übte, überwiegend als berechtigt gilt, fühlen sich heute nur wenige seinem kommunikationstheoretischen Begründungsprogramm verpflichtet. Die von mir hinterfragte Prämisse vom Originalmodus verständigungsorientierten Sprechens und die fehlgeschlagene Ableitung des Moralprinzips sind dabei nur zwei Probleme unter vielen. Wie analytisch geschulte Autoren (Keuth 1993, 263-346; Lumer 1997; Steinhoff 2006; Tugendhat 1993) minutiös gezeigt haben, stößt man auf nahezu jeder Ebene auf angreifbare Prämissen, nicht gedeckte Schlüsse und petitiöse Argumentationen. Im Laufe der Zeit verfestigte sich so der Eindruck, dass einzelne Korrekturen die Sache nicht mehr retten können; das diskursethische Begründungsprogramm gilt gemeinhin als gescheitert. Darüber hinaus wurde die Praxis-Ferne der Diskursethik bemängelt. Aufgrund ihres formalistisch-prozeduralen Zuschnitts scheide sie als inhaltlicher Impulsgeber sozialer Bewegungen aus (Reese-Schäfer 2001, 75 ff.), deren Proteste und Widerstände, so Honneth (2000), sich ohnehin nur hermeneutisch erschließen lassen. Für diese Einschätzung gibt es sogar Anhaltspunkte bei Habermas selbst: „Die *moralischen* Alltagsintuitionen bedürfen der Aufklärung des Philosophen nicht. [...] Die philosophische Ethik hat eine aufklärende Funktion allenfalls gegenüber den Verwirrungen, die sie selbst im Bewußtsein der Gebildeten angerichtet hat.“ (Habermas 2009, 101) Die lebensweltliche Normativität ist nicht angewiesen auf moralphilosophische Belehrung; die Diskursethik dient lediglich der intellektuellen Selbstkorrektur (kritisch hierzu Apel 1989). Vergleichbares ließe sich auch über die dualistische Sozialtheorie von Habermas sagen. Insbesondere sein systemtheoretisches Verständnis der kapitalistischen Ökonomie, die sich über das neutrale Steuerungsmedium Geld reguliere und der technisch effizienten Güterproduktion diene, gilt vielen als reduktionistisch und affirmativ (u.a. Elbe 2015). Das Verhältnis zu Habermas ist folglich ambivalent: Auf der einen Seite folgt man ihm überwiegend in seiner Diagnose, dass die soziale Wirklichkeit nicht-instrumentelle Sinnhorizonte bereithält, auf der anderen Seite hält man sein ethisches Begründungsprogramm und sein Ökonomieverständnis für wenig überzeugend und politisch belanglos. Dies erklärt den Erfolg der sogenannten *internen Kritik*. Wenn Habermas doch gezeigt hat, dass normative Ansprüche lebensweltlich bereits verankert sind, warum soll man dann nicht *direkt* an sie anknüpfen, d.h. sie zur Kritik persistierender Verdinglichungspathologien in Anspruch nehmen? Anstatt, wie Habermas gegenüber Apel, den Begründungsanspruch nur zu relativieren, könne man auf den problembeladenen Umweg eines moralphilosophischen Begründungsprogramms auch gänzlich verzichten. Die normativen Prinzipien ergeben sich dann im Zuge einer Rekonstruktion der modernen Sozial- und Theoriegeschichte ganz unmittelbar – und lassen sich womöglich noch in den Bereichen nachweisen, die Habermas als Subsysteme strategischen Handelns, d.h. als nicht-normativ begriff (Honneth 2011).

Die pragmatischen Vorzüge dieses Ansatzes, darauf hat Rahel Jaeggi (2014, 261 ff.) hingewiesen, sind jedoch teuer erkauft. Interne Kritik weist nur ein geringes Emanzipationspotential auf, weil sie, strukturell konservativ, auf anerkannte Normen setzt, denen lediglich die Praxis nicht entspricht. Interne Kritik macht sich zudem auf gefährliche Weise von ihrem Gegenstand abhängig: Sie setzt auf Werte und Normen, die historisch kontingent sind. Dies war schon für Habermas der Grund, nach *Strukturwandel der Öffentlichkeit* auf eine formalpragmatische Begründungsstrategie umzuschalten. Jaeggi zieht jedoch eine andere Konsequenz. Anstatt zum externen Kritikmodus zurückzukehren, schlägt sie vor, den internen Ansatz zu modifizieren. Anknüpfungspunkt der von ihr als *immanent* bezeichneten Kritik dürften (a) nicht bloß kontingente Normen, sondern sozialformationsspezifische Widersprüche sein, die (b) vermittelt über Krisen über sich selbst hinaustreiben, d.h. die bestehende Gesellschaft transzendieren. Das Problem, dass der Kapitalismus keine funktionalen Krisen aufweist, die seine Existenz infrage stellen, sondern die ökonomischen Krisen vielmehr die nachträgliche (Steuerungs‑)Funktion einer ungesteuerten Wirtschaft darstellen, glaubt Jaeggi dadurch umgehen zu können, dass die Krisen keine rein funktionalen Krisen seien, sondern auch eine normative Seite aufweisen. „Ein ‚praktischer Widerspruch‘ ist […] dadurch gekennzeichnet, dass in einem gesellschaftlichen Prozess Hemmnisse oder Krisen auftreten, die in beiden Hinsichten problematisch sind: *Etwas funktioniert nicht gut, und es ist nicht gut, wie es funktioniert*.“ (Jaeggi 2014, 306) Die Aussage, es sei nicht gut, wie der Kapitalismus funktioniert, setzt jedoch ein Verständnis des Guten voraus. Nur ist erstens unklar, wo ein solches Verständnis herkommen soll – denn Jaeggi will weder, wie die interne Kritik, auf Normen der sozialen Konvention zurückgreifen, noch, wie die externe Kritik, universalistische Normen auf dem Reißbrett entwerfen (vgl. Khurana 2019, 122) –, und zweitens erweckt auch der um die normative Dimension erweiterte Krisenbegriff den Eindruck des revolutionstheoretischen *wishful thinking*, unterstellt er doch wie sein funktionalistischer Vorgänger eine systemsprengende Dynamik (vgl. Egger 2018). Hinter beiden Problemen scheint sich mir eine Prämisse von Horkheimer und Adorno zu verbergen, die in der Diskussion meist von der geschichtsphilosophischen Problematik der *Dialektik der Aufklärung* überschattet wurde: die u.a. auch von Freyenhagen (2013) vertretene These des metaethischen Negativismus, dass wir qua somatischer Reaktion lediglich wissen können, was ethisch verwerflich ist, aber darum noch über kein positives Wissen um das Gute oder Rechte verfügen (siehe hierzu kritisch Ellmers 2020, 2021).

Einen wichtigen Kontrapunkt zur genealogischen, internen, immanenten und negativistischen Kritik bildet die Rechtfertigungstheorie von Rainer Forst. Getragen ist sie von der Idee, die Probleme der Diskursethik dadurch zu beheben, dass man sie auf ein solides kantisches Fundament stellt.

Forst unterscheidet zunächst zwischen *rationaler Begründung* (der Mittelwahl für abzuwägende subjektive Ziele) und *vernünftiger Rechtfertigung* (mittels intersubjektiv vertretbarer Gründe), wobei er letztere in ethische und moralische Rechtfertigung differenziert (vgl. Forst 2007, 25 ff.). Die *ethische* Rechtfertigung vollzieht sich stets im Rahmen eines partikularen Wertekanons. So kann man sich die Frage stellen, ob eine Handlung mit dem Selbstbild (den individuellen Überzeugungen) vereinbar ist, man kann über die Verpflichtungen nachdenken, die man als Teil einer besonderen Gemeinschaft zu erfüllen hat, oder man kann zusammen mit anderen das Ethos der Gemeinschaft thematisieren. Bei der moralischen Rechtfertigung hingegen geht es um basale Pflichten, denen eine „*kategorisch* bindende Kraft innewohnt“ (ebd., 29): Die Moral umfasst das (und *nur* das), was Menschen einander unbedingt schulden, weil sie Menschen sind (vgl. ebd., 76, 305). Sie hat ihren eigenen Geltungsbereich – und dass sie ihn hat, setzt „eine gewisse Vorstellung menschlicher Freiheit und Vernunft voraus“ (ebd., 76). Mit der kantischen These einer Autonomie der Moral, die in moralischer Autonomie gegründet ist – d.h. in dem Vermögen, seine Handlungen an Grundsätzen auszurichten, die sich als universell gültig rechtfertigen lassen (vgl. ebd., 31) –, distanziert sich Forst von Honneths und Jaeggis These, Gesellschaftskritik könne lediglich intern bzw. immanent geübt werden (vgl. Forst 2015a, 13 ff.). Während beide davon ausgehen, an bereits institutionalisierte Normen anknüpfen zu müssen, weil wir ethisch derart radikal situierte Wesen sind, dass jede universalistische Ethik einer Selbsttäuschung gleichkommt, setzt Forst auf die „transzendierende Kraft der Vernunft“: „Die eingelebte Sittlichkeit ist der *Gegenstand* der Kritik, nicht ihr *Grund* oder ihre *Grenze*.“ (Forst 2015a, 16) Wir benötigen einen letzten, universell gültigen, formalen, aber trotzdem zu substantiellen Unterscheidungen fähigen Grundsatz, um entscheiden zu können, „*welche* immanenten Prinzipien es wert sind, Grundlage der Kritik zu sein und welche nicht“ (Forst 2017, 61).

Diesen Grundsatz gewinnt Forst über das *Rechtfertigungsprinzip*. Es besagt, dass „normative Antworten auf praktische Fragen auf genau die Weise zu rechtfertigen sind, auf die ihr Geltungsanspruch verweist.“ (Forst 2007, 32) Angewendet auf *moralische* Kontexte besagt dies, dass Normen, die beanspruchen, reziprok und allgemein zu gelten, auch reziprok und allgemein rechtfertigbar sein müssen (vgl. Forst 2007, 33). Dies erinnert nicht von ungefähr an den Gedanken von Habermas, dass jede Person, die einen Richtigkeitsanspruch erhebt, die formalprozeduralen Bedingungen unterstellen muss, durch die allein sich der kritisierbare Anspruch erst einlösen ließe; wer ernsthaft für eine Norm argumentiert, antizipiert den herrschaftsfreien Diskurs, dessen Regeln zwar einerseits kontrafaktisch, andererseits aber auch unvermeidlich und darum eben doch faktisch wirksam sind, d.h. als notwendige Idealisierungen „über die Realität hinausschießen.“ (Habermas 2016, 808) Kurz: Der durch die Anwendung des Rechtfertigungsprinzips gewonnene Moralgrundsatz reziprok-allgemeiner Rechtfertigung übernimmt die Rolle von U und D bei Habermas. Und dennoch gibt es Unterschiede. So unternimmt Forst nicht den Versuch, aus allgemeinen Präsuppositionen der argumentativen Rede Diskursregeln zu entwickeln, um aus diesen wiederum moralische Maßstäbe abzuleiten. Forst geht keine kommunikationstheoretischen Umwege, die sich zudem als Sackgassen erwiesen haben; er nimmt vielmehr eine Abkürzung: Er schließt direkt vom universellen Geltungsanspruch einer Norm auf die diskursiven Bedingungen seiner Einlösung. Die Kriterien, die darüber befinden sollen, ob eine Norm eine moralische Norm ist (Reziprozität und Allgemeinheit), entnimmt Forst unmittelbar dem moralischen Geltungsanspruch. „An die Rechtfertigungspraxis werden keine […] kontextfremden Kriterien herangetragen, sondern nur die, die im Anspruch [...] selbst enthalten sind.“ (Forst 2007, 32)

Welchen Geltungsanspruch erheben Normen im Kontext der Moral? Sie erheben Forst zufolge insofern einen *kategorischen* Anspruch, als jedes Mitglied der moralischen Gemeinschaft von jedem anderen Mitglied der moralischen Gemeinschaft die Rechtfertigung und Erfüllung der in der Norm definierten Pflicht verlangen kann. Der Autor einer Norm darf demnach keine Forderungen stellen, die er selbst nicht zu erfüllen bereit wäre (Reziprozität der Inhalte), er darf seine Wertvorstellungen und Bedürfnisse nicht auf andere projizieren und die Pflicht auch nicht auf bestreitbaren Prämissen gründen (Reziprozität der Gründe), und schließlich darf er die Rechtfertigungsgemeinschaft nicht willkürlich eingrenzen, d.h. sie muss alle von einer Handlung oder Handlungsnorm Betroffenen umfassen (Allgemeinheit) (vgl. ebd., 306). „Diese beiden Kriterien zusammengenommen verleihen moralischen Personen ein grundlegendes, wenngleich qualifiziertes *Vetorecht* – eben das basale Recht auf Rechtfertigung.“ (ebd.) Indem Forst herausstellt, dass der Mensch ein Grund-Recht auf und eine Grund-Pflicht zur Rechtfertigung hat, votiert er für einen „more substantive Kantianism than we find in Habermas“:

I defend a Kantian approach in the sense that I think that, with respect to the right of justification, practical reason and morality are united in criticizing the unwillingness to provide adequate justifications for one’s morally relevant actions as a failure of reason and of morality at the same time. Discoursetheoretical Kantianism without a categorical imperative, by contrast, would only be able to reconstruct the discursive rules of the moral language game and then look for the motivation to engage in that game in the non-moral realm, for the moral realm would only begin once we „want“ to play that game—and that strikes me as insufficient. (Forst 2015b, 823)

In einen Diskurs über kategorische Rechte und Pflichten einzutreten, ist selbst kategorisch geboten, d.h. keine Frage der Willkür, sondern der „Würde“ (Forst 2007, 10) des autonomen Rechtfertigungswesens Mensch. Im Diskurs erweist sich die Moralität von Normen und Handlungen^15^ nun daran, ob sie ihrem eigenen Anspruch gerecht werden: und zwar durch die Angabe von Gründen, die – diese Formulierung übernimmt Forst von T. Scanlon – vernünftigerweise nicht zurückgewiesen werden können (wobei ein Grund genau dann vernünftigerweise nicht zurückgewiesen werden kann, wenn er die Kriterien der Reziprozität und Allgemeinheit erfüllt). Werden gegen Normen und Handlungen, für die gute (d.h. reziprok-allgemeine) Gründe vorgebracht wurden, lediglich partikulare Einwände geltend gemacht, können sie, zumindest vorläufig, als gerechtfertigt gelten.

Deutlich wird hier eine doppelte Stoßrichtung: Indem Forst erstens betont, dass der Geltungsanspruch einer Norm *diskursiv* einzulösen ist, schließt er sich Habermas’ Kritik an Kants monologischer Normenrechtfertigung an (vgl. ebd., 35). Nur wenn die Betroffenen selbst zu Wort kommen, wird „die stillschweigende Privilegierung je meiner Sicht der Dinge verhindert“ (Habermas 1991b, 157). Da sich die Diskursethik allerdings dem Vorwurf ausgesetzt sieht, sie erhebe den faktischen Konsens zum Moralitätskriterium, betont Forst zweitens, dass wir mit Hilfe der Kriterien Reziprozität und Allgemeinheit auch bei Dissensen ein begründetes Urteil über die Moralität einer Norm fällen können.

Eingehend diskutieren kann ich diese Weichenstellung hier nicht mehr. Bereits jetzt lässt sich jedoch sagen, dass mit Forsts Rechtfertigungstheorie entscheidende Schritte zurück getan sind, um die Debatte über den normativen Maßstab der Gesellschaftskritik produktiv fortzuführen. „Dabei muss festgehalten werden, dass keine Theorie als ‚kritische‘ auftreten kann, die sich ihres Vernunftbegriffs nicht explizit vergewissert und diesen nicht auch selbst der Kritik unterwirft.“ (Forst 2015a, 12). Here weg go!

## Siglen

### DdA

Adorno, Theodor W. / Horkheimer, Max. 1997. *Dialektik der Aufklärung. Philosophische Fragmente*. In *Gesammelte Schriften*, Bd. 3. Darmstadt: Wissenschaftliche Buchgesellschaft.

### TKH

Habermas, Jürgen. 1981. *Theorie des kommunikativen Handelns. Band 1. Handlungsrationalität und gesellschaftliche Rationalisierung*. Frankfurt am Main: Suhrkamp.

### SGS

Habermas, Jürgen. 1988/2009. Handlungen, Sprechakte, sprachlich vermittelte Interaktionen und Lebenswelt. In *Philosophische Texte, Bd. 1, Sprachtheoretische Grundlegung der Soziologie*, 197–242. Frankfurt am Main: Suhrkamp.

### ZK

Horkheimer, Max (1991), *Zur Kritik der instrumentellen Vernunft*. In *Gesammelte Schriften*, Bd. 6, Frankfurt am Main: Fischer.

## Footnotes

1 „Furchtbares hat die Menschheit sich antun müssen, bis das Selbst, der identische, zweckgerichtete, männliche Charakter des Menschen geschaffen war.“ (DdA 50).

2 Die der sozialen Herrschaft zugrundeliegende instrumentelle Rationalität „ist im Grund gar nichts anderes als die auf den Menschen übertragene […] Ausbeutung der Natur.“ (Adorno 2001, 27).

3 Vereinzelt argumentiert Horkheimer sogar genau in diesem Sinne: zum Beispiel wenn er die „totalitäre“ Instrumentalisierung der Natur auf die besondere Qualität der „Beziehungen zwischen Menschen“ (ZK 119), d.h. auf die Produktions*verhältnisse* zurückführt. „Der Kriegszustand unter den Menschen in Krieg und Frieden ist der Schlüssel für die Unersättlichkeit der Gattung“ (ebd.). Konsistent ist Horkheimer in dieser Frage jedoch nicht. Mal ruft das vorgängige Mensch-Natur-Verhältnis instrumentelles Denken hervor, mal sind die Produktivkräfte Motor der Entwicklung (siehe unten) und dann sind wieder die Produktionsverhältnisse das Bedingende.

4 So heißt es im Protokoll einer Seminardiskussion von 1936 zur Marxschen Wertlehre: „Die logischen Voraussetzungen der Marxschen Methode bezeichnen zugleich die historischen Voraussetzungen des Gegenstands. Einfache Warenproduktion ist nicht bloß eine begriffliche Vereinfachung, sondern auch eine geschichtliche Vorstufe. […] Diese Durchdringung logischer und historischer Kategorien ist ein wichtiges Charakteristikum der dialektischen Methode.“ (Horkheimer 1985a, 400).

5 Der „Prozess der Rationalisierung“ sei „nicht länger das Ergebnis der anonymen Kräfte des Marktes“, vielmehr werde „im Bewusstsein einer planenden Minderheit über ihn befunden“ (108). Anonyme Herrschaft sei wieder durch personale Herrschaft abgelöst worden. Ausschlaggebend sind nicht länger systemische Imperative, sondern die Kommandos der Rackets: verschworener Machtcliquen aus Ökonomie, Politik, Bürokratie und Militär, die die Gesellschaft nach ihrem Willen und zu ihrem Vorteil lenken (siehe hierzu Horkheimer 1985b, c).

6 Eine Perspektive, die für Freud wenig Sinn ergeben hätte. Betont er doch schon in der ersten Fassung seiner Trieblehre, dass mit der Entwicklung des der Außenwelt zugewandten Ichs oder Real-Ichs das Lustprinzip *nicht *außer Kraft gesetzt ist. „In Wirklichkeit bedeutet die Ersetzung des Lustprinzips durch das Realitätsprinzip keine Absetzung des Lustprinzips, sondern nur eine Sicherung desselben. Eine momentane, in ihren Folgen unsichere Lust wird aufgegeben, aber nur darum, um auf dem neuen Wege eine später kommende, gesicherte zu gewinnen.“ (Freud 2000b [1911]) Auch wenn Freud seine Trieblehre noch modifizieren wird (insbesondere durch die Entgegensetzung von Lebens- und Todestrieben), so bleibt das ursprüngliche Verhältnis von Realitäts- und Lustprinzip unverändert. In der Spätschrift *Neue Folge der Vorlesungen zur Einführung in die Psychoanalyse* bezeichnet er das Ich prägnant „als Teil des Es“ (Freud 2000c [1932], 512). „Im ganzen muß das Ich die Absichten des Es durchführen, es erfüllt seine Aufgabe, wenn es die Umstände ausfindig macht, unter denen diese Absichten am besten erreicht werden können.“ (ebd., 514) Sehr eindrücklich ist auch die Allegorie von Pferd und Reiter, die Freud verwendet, um das Verhältnis von Ich und Es zu verdeutlichen: „Das Pferd gibt die Energie für die Lokomotion her, der Reiter hat das Vorrecht, das Ziel zu bestimmen, die Bewegung des starken Tieres zu leiten. Aber zwischen Ich und Es ereignet sich allzu häufig der nicht ideale Fall, daß der Reiter das Roß dahin führen muss, wohin es selbst gehen will.“ (ebd.).

7 Ähnlich Adorno: „daß es irrationale Ausbrüche gibt, das steht ja außer Frage, nur muß man das damit einschränken, daß die sogenannten Aus- und Aufbrüche irrationaler oder urtümlicher Mächte in unserer Zeit fast immer manipuliert waren und fast immer im Sinn der Durchsetzung von Herrschaft […] standen und insofern selber in den Zug rationaler Beherrschungstechniken fallen. Das hat man mit besonderer Stringenz natürlich am Nationalsozialismus studieren können.“ (Adorno 2001, 24).

8 Um Missverständnisse zu vermeiden: Erstens bin ich nicht der Ansicht, dass eine Theorie der kapitalistischen Moderne schon hinreichend wäre, um den Antisemitismus in der Moderne zu erklären. Zwar ist die Shoa nicht nur eine Fortsetzung der langen Geschichte des religiösen Antijudaismus, ohne diese Geschichte (siehe hierzu Maccoby 2019) lässt sie sich aber auch nicht verstehen. Meine Kritik an den Folgen, die der instrumentalisierungs- und zivilisationskritische Ansatz für das Verständnis von Antisemitismus hat, beruht also nicht ihrerseits auf modernitätstheoretischen Verengungen. Zweitens möchte ich keineswegs in Abrede stellen, dass die erste Generation der Kritischen Theorie Pionierarbeit für die Antisemitismusanalyse geleistet hat. Diese Pionierarbeit war möglich, weil sich ihr Denken nicht in den von mir kritisierten Theoremen erschöpfte, sondern sie mit der (primär von Erich Fromm entwickelten) analytischen Sozialpsychologie auch über produktivere theoretische Ressourcen verfügte.

9 Deckungsgleich: Marcuse (1980 [1938], 69).

10 Die Unterscheidung von Lokution, Illokution und Perlokution geht auf John L. Austin (1976, 94-121) zurück. Ein lokutionärer Akt besteht in dem Hervorbingen einer sprachlichen Lautkette, die den grammatischen Regeln einer bestimmten Sprache folgt und sich auf etwas in der Welt bezieht. Für einen illokutionären Akt ist entscheidend, dass durch den lokutionären Akt eine Handlung vollzogen wird. Der Sprecher kann den Hörer beispielsweise um etwas bitten, ihm etwas empfehlen oder ihm drohen. Der perlokutionäre Akt wiederum besteht ist dem Erzielen einer Wirkung, die über den illokutionären Akt hinausgeht. Beispielweise wird durch den lokutionären Akt „Du baust noch einen Unfall, wenn Du nicht langsamer fährst“ der illokutionäre Akt der Warnung vollzogen und dadurch eine beabsichtigte oder unbeabsichtigte Wirkung erzielt (z.B. der Fahrer fährt langsamer).

11 Contre cœur bestätigt dies Habermas sogar selbst: „Die Rationalität des Handelns bemißt sich […] daran, ob der Aktor dieses Ergebnis aufgrund der vorsätzlich gewählten und eingesetzten Mittel erzielt hat“ (Habermas 1997, 263). „Die Rationalität des verständigungsorientierten Sprachgebrauchs hängt […] davon ab, ob die Sprechhandlungen so verständlich und akzeptabel sind, dass der Sprecher damit illokutionäre Erfolge erzielt“ (ebd.). Die zweite Definition ist ganz offensichtlich nur eine Anwendung der ersten Definition auf den besonderen Fall verständigungsorientierter Behauptungen.

12 Dass der Bankräuber keine *Gründe* angibt, sondern die Geisel *erschießt* (also handelt), folgt daraus, dass sich die Wahrhaftigkeit der Selbstdarstellung eines Sprechers gar nicht anders als durch konsistente Handlungen einlösen lässt. Verständigungsorientierte Sprechakte bilden hier keine Ausnahme: „Der Sprecher kann allenfalls in der Konsequenz seiner Handlungen beweisen, ob er das Gesagte auch wirklich gemeint hat. Die Wahrhaftigkeit von Expressionen läßt sich nicht *begründen*, sondern nur *zeigen*“ (TKH 69; ähnlich Habermas 2009, 51).

13 Eine der wenigen Ausnahmen ist Stoetzler (2019).

14 Ein vergleichbarer Nietzsche-Zugriff findet sich bei Flügel-Martinsen (2017): Nicht das *Be*gründen, sondern das *Ent*gründen sei die Stärke der Genealogie.

15 Das Moralprinzip dient bei Forst nicht nur der Rechtfertigung von Normen, sondern auch von Handlungen in konkreten Situationen. Siehe hierzu Forst (2007, 34) und Forst (2015b, 825 f.).
